# Modulation of *Lactobacillus plantarum* Gastrointestinal Robustness by Fermentation Conditions Enables Identification of Bacterial Robustness Markers

**DOI:** 10.1371/journal.pone.0039053

**Published:** 2012-07-03

**Authors:** Hermien van Bokhorst-van de Veen, I-Chiao Lee, Maria L. Marco, Michiel Wels, Peter A. Bron, Michiel Kleerebezem

**Affiliations:** 1 TI Food and Nutrition, Wageningen, The Netherlands; 2 NIZO Food Research, Ede, The Netherlands; 3 Laboratory of Microbiology, Wageningen University and Research Centre, Wageningen, The Netherlands; 4 Host-Microbe Interactomics, Wageningen University and Research Centre, Wageningen, The Netherlands; 5 Centre for Molecular and Biomolecular Informatics, Radboud University Medical Centre, Nijmegen, The Netherlands; 6 Kluyver Centre for Genomics of Industrial Fermentation, Delft, The Netherlands; University of Groningen, Netherlands

## Abstract

**Background:**

Lactic acid bacteria (LAB) are applied worldwide in the production of a variety of fermented food products. Additionally, specific *Lactobacillus* species are nowadays recognized for their health-promoting effects on the consumer. To optimally exert such beneficial effects, it is considered of great importance that these probiotic bacteria reach their target sites in the gut alive.

**Methodology/Principal Findings:**

In the accompanying manuscript by Bron *et al.* the probiotic model organism *Lactobacillus plantarum* WCFS1 was cultured under different fermentation conditions, which was complemented by the determination of the corresponding molecular responses by full-genome transcriptome analyses. Here, the gastrointestinal (GI) survival of the cultures produced was assessed in an *in vitro* assay. Variations in fermentation conditions led to dramatic differences in GI-tract survival (up to 7-log) and high robustness could be associated with low salt and low pH during the fermentations. Moreover, random forest correlation analyses allowed the identification of specific transcripts associated with robustness. Subsequently, the corresponding genes were targeted by genetic engineering, aiming to enhance robustness, which could be achieved for 3 of the genes that negatively correlated with robustness and where deletion derivatives displayed enhanced survival compared to the parental strain. Specifically, a role in GI-tract survival could be confirmed for the *lp_1669*-encoded AraC-family transcription regulator, involved in capsular polysaccharide remodeling, the penicillin-binding protein Pbp2A involved in peptidoglycan biosynthesis, and the Na^+^/H^+^ antiporter NapA3. Moreover, additional physiological analysis established a role for Pbp2A and NapA3 in bile salt and salt tolerance, respectively.

**Conclusion:**

Transcriptome trait matching enabled the identification of biomarkers for bacterial (gut-)robustness, which is important for our molecular understanding of GI-tract survival and could facilitate the design of culture conditions aimed to enhance probiotic culture robustness.

## Introduction

According to the world health organization (WHO) probiotics are defined as live microorganisms which, when administered in adequate amounts, confer a health benefit on the host [1]. The most widely applied probiotic strains belong to the genera *Lactobacillus* and *Bifidobacterium* [2], [3]. Probiotics are most commonly provided as freshly fermented food products, non-fermented food products to which probiotics are added, or as dried bacterial preparations [3], [4]. The viability of bacteria is considered an important trait for probiotic functionality, justifying the interest to unravel the mechanism(s) involved in gastrointestinal (GI)-tract survival at the molecular level [5], [6], [7], [8].

During passage through the GI-tract, probiotics encounter several stresses including acidity in the stomach which may reach a pH as low as 1 during fasting [7]. This low extracellular pH affects the proton motive force of the bacterial cells, thereby disrupting the energy supply required for processes such as membrane transport [9]. In addition, lower intracellular pH values caused by acidic conditions may inhibit specific pathways by damaging acid-sensitive associated enzyme functions [9]. After stomach passage probiotic strains reach the small intestine, where bile acids act primarily as a surfactant that can disrupt bacterial membranes [10] and damage macromolecules such as RNA and DNA through the generation of free oxygen radicals [11]. Moreover, protonated bile acids can freely pass bacterial cell membranes and release protons intracellularly which might lead to lowering of the intracellular pH, analogous to acid stress [9].

Among the lactobacilli, *Lactobacillus plantarum* is encountered in a plethora of fermentations, ranging from vegetables to dairy, meat and sourdough [12], [13]. *L. plantarum* is also frequently encountered as a natural inhabitant of the GI-tract of several mammals, including humans [14]. In addition, *L. plantarum* NCIMB8826 was demonstrated to effectively survive passage of the human stomach, reached the ileum in high numbers, and was detected in the colon [15]. A single colony isolate of this strain (designated *L. plantarum* strain WCFS1) was the first *Lactobacillus* strain of which the full genome sequence was published [16]. Subsequently, sophisticated bioinformatics tools were developed for this LAB strain, including an advanced genome annotation [17], genome-based metabolic models [18], as well as effective mutagenesis tools [19]. This enables the molecular investigation of gene-regulatory mechanisms underlying the observed GI-tract persistence of *L. plantarum* WCFS1.

The availability of full genome sequences has also enabled the exploration of genomic diversity among *L. plantarum* strains and its association to differential phenotypes [13], [20], [21], [22], [23]. To enable the identification of genes of which the relative expression level is correlated to the phenotype of interest, we recently developed a complementary transcriptome-phenotype matching strategy for *L. plantarum* (see accompanying paper by Bron *et al.*). Here, we employed this fermentation genomics platform to correlate transcriptome data to GI-tract survival. These correlations led to the identification of 13 candidate effector molecules for GI-tract persistence. A subsequent gene deletion strategy established a definite role in GI-tract persistence for the AraC-family transcription regulator encoded by *lp_1669*, the penicillin-binding protein Pbp2A involved in peptidoglycan biosynthesis, and the Na^+^/H^+^ antiporter NapA3.

## Results

### Gastric Acidity is a Critical Determinant of *L. plantarum* Survival

**Figure 1:**
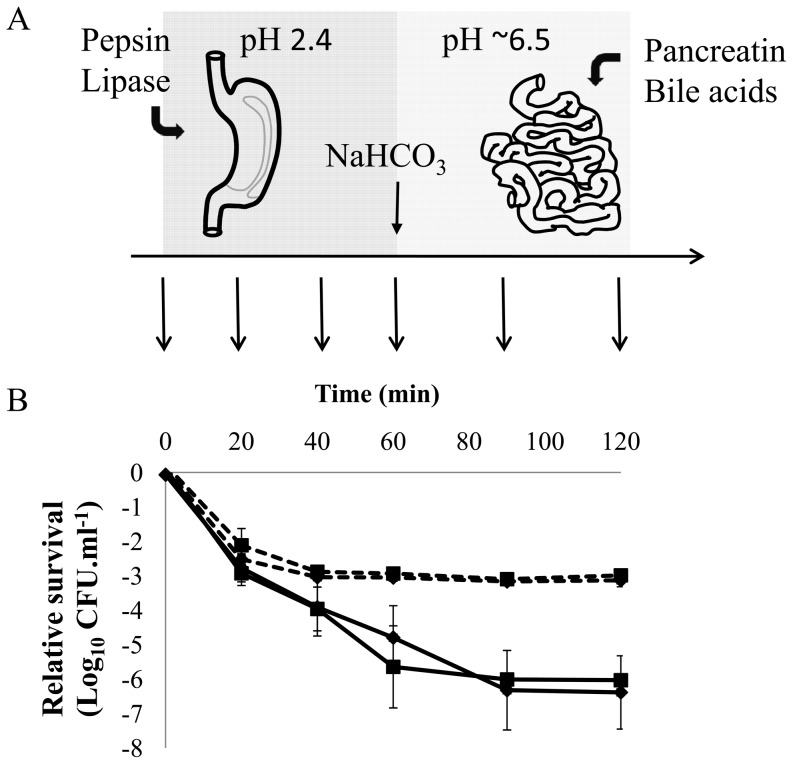
Relative survival of *L. plantarum* cells, subjected to an upper gastrointestinal-tract mimicking assay. *L. plantarum* WCFS1 cultures were grown aerobically at 28°C in 2×CDM containing normal acid concentration, at a pH of 5.8 and without NaCl. The cultures were harvested at mid-exponential phase (OD_600_ = 1.0) and subjected to an upper GI-tract mimicking assay (A): After 60 min incubation in gastric juice containing pepsin and lipase at a pH of 2.4 (logarithmic cells) or 2.3 (stationary cells), cultures were neutralized with NaHCO_3_ and pancreatic juice containing pancreatin and bile acids was added and incubation continued for 60 min (see materials and methods for details). Preceding and during incubation, samples were taken for CFU determination (aligned arrows). Panel B shows the relative survival of two independent cultures in logarithmic phase (solid lines) and stationary phase (dashed lines) during the GI-tract mimicking assay. Input (CFU determination immediately prior to the GI assay) is set at 0 Log_10_ CFU ml^−1^, data presented are averages of technical sextuplicates (+standard deviation).

An *in vitro* assay was developed that allows high-throughput assessment of bacterial GI-tract survival (fig. 1A). Two independent reference *L. plantarum* WCFS1 cultures that were harvested during logarithmic phase of growth (OD_600_ = 1.0) displayed a 6-log decrease in CFU·ml^−1^ in the GI-tract assay (fig. 1B). The survival curves of these reference cultures demonstrated the major impact on survival exerted by gastric juice on *L. plantarum* viability and the relatively minor effect of the conditions which resembled the small intestine (fig. 1B). This differential effect on survival during the two stages within the GI-tract assay was consistently observed for all cultures tested, irrespective of the fermentation conditions applied or the growth phases from which bacterial cells were harvested.

The strongest determinant in the loss of survival during the gastric juice treatment appeared to be pH. For screening log-phase cells of *L. plantarum,* a pH of 2.4 was used for cells, as lowering or increasing of the gastric juice pH by 0.1 pH unit resulted in death or survival of almost all cells, respectively (over 7-log differences, data not shown). *L. plantarum* cells harvested at the stationary phase of growth consistently displayed a higher tolerance to the gastric juice treatment, which is exemplified by their higher survival rate in the GI-tract assay when a reduced pH of 2.3 was used (fig. 1B) at which the cells harvested from the logarithmic phase of growth were nearly all killed within 60 minutes of incubation.

### Fermentation-enhanced Digestive Tract Survival

**Figure 2:**
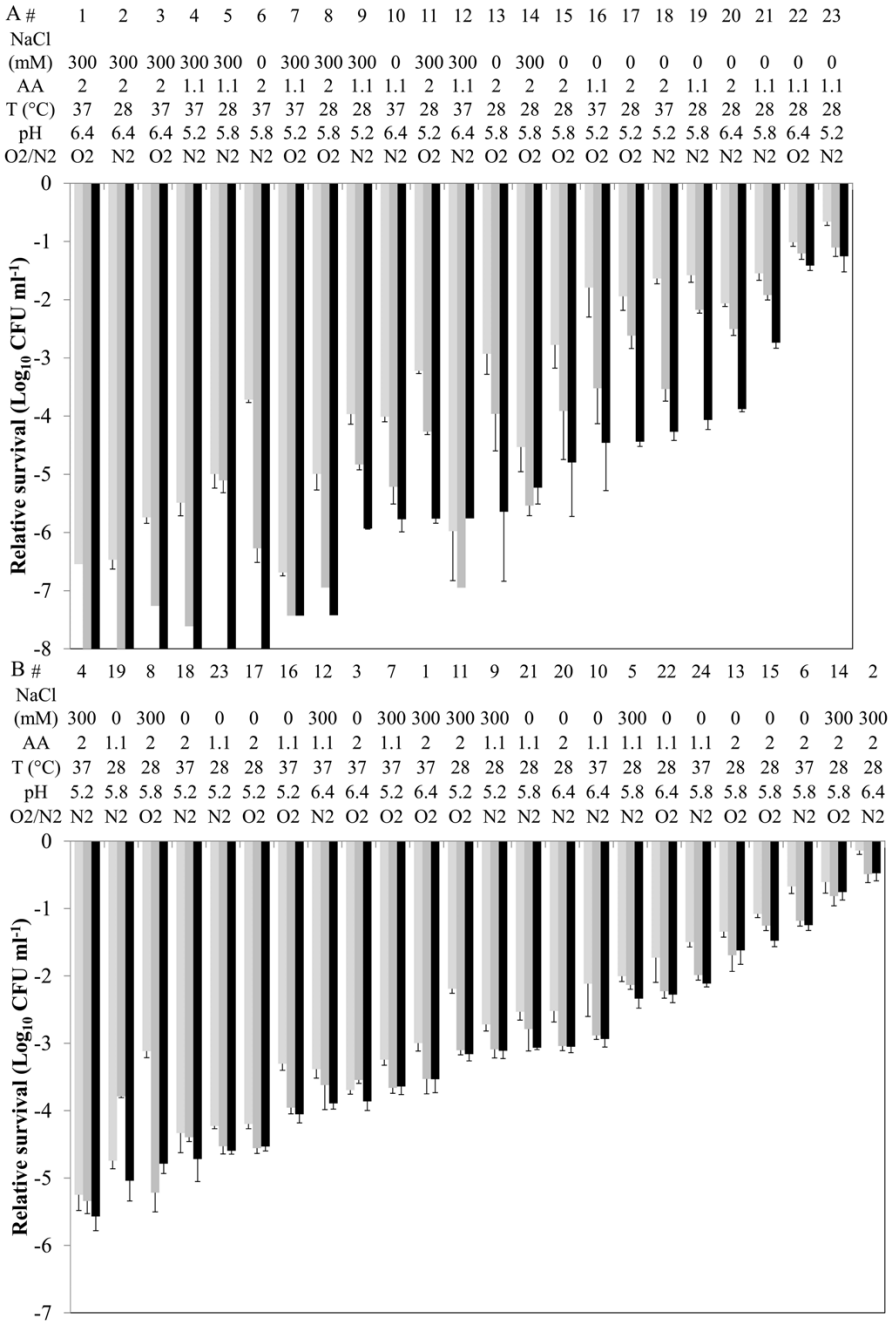
Relative GI-tract survival of differently grown *L. plantarum* WCFS1. Log_10_ CFU ml^−1^ determination of *L. plantarum* WCFS1 in logarithmic phase (A) and stationary phase (B) after 20 (light grey), 40 (dark grey), and 60 min (black) gastric juice incubation. Input is set at 0 Log_10_ CFU ml^−1^, #  =  fermentation number, cultures were grown in 2× CDM with (300 mM) or without (0) NaCl; with normal amino acid concentration (2) or reduced (1.1); at 28°C or 37°C; medium buffered at a pH of 5.2, 5.8, or 6.4; and aerobically (O_2_) or anaerobically (N_2_). Data presented are averages of technical sextuplicates (+ standard deviation).

We examined the effects of different growth conditions on *L. plantarum* WCFS1 GI-tract survival by applying samples derived from the fermentation-genomics platform described in the accompanying paper by Bron *et al*. to our *in vitro* GI-tract assay. The results demonstrate that fermentation conditions used to culture *L. plantarum* WCFS1 conferred a profound influence on the GI-tract survival. Variable fermentation conditions resulted in major differences (a reduction of 7 logs for the logarithmic population and 5 logs for stationary cells) in *L. plantarum* WCFS1 survival after incubation in gastric juice (fig. 2). Notably, survival of cultures grown in different fermentation conditions strongly exceeded the levels of variation in survival observed in independent GI-tract assays (fig. 1B).

**Figure 3:**
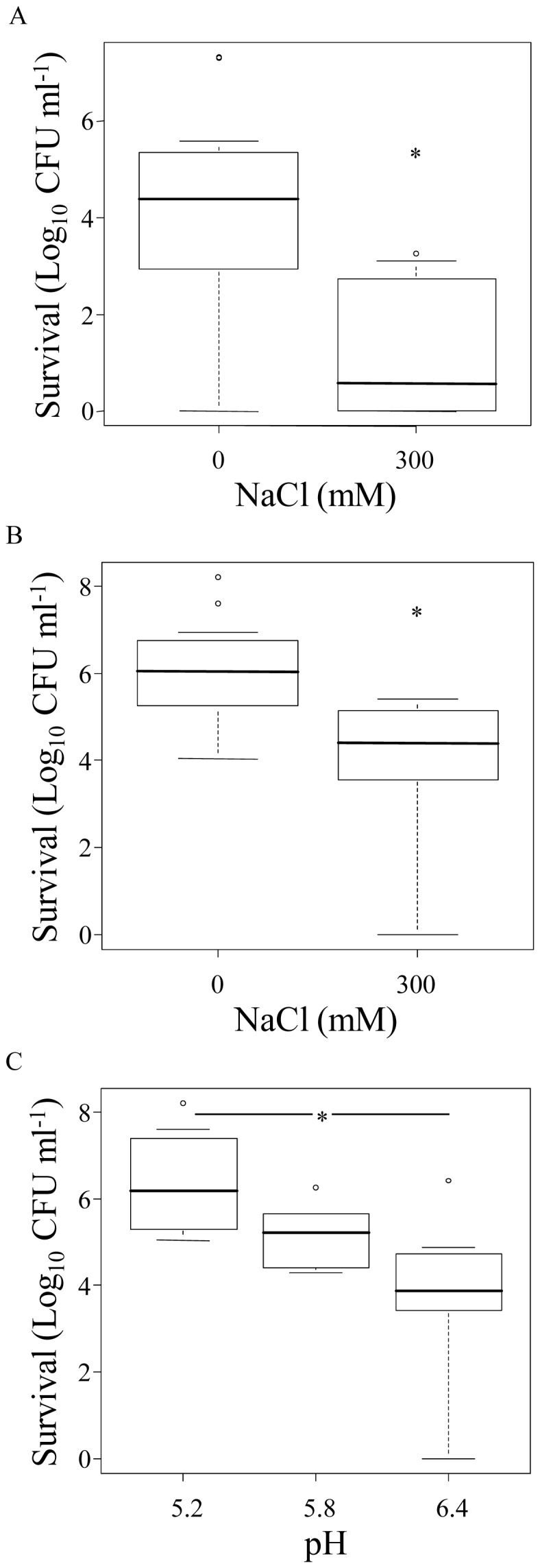
Effect of medium components on GI-tract survival of *L. plantarum* WCFS1. Box plots of NaCl and 60 min GI-tract survival of logarithmically (A) and stationary (B) grown cultures and of pH and 60 min GI-tract survival of stationary cells (C). Results are based on data from all fermentations used in this study (see fig. 2A). * *P*-value <0.05 compared with 0 mM NaCl (A and B) or pH 6.4 (C).

To identify the fermentation conditions that significantly affected the survival rate in the simulated GI-tract conditions, a Mann-Whitney U test-based correlation analysis was performed in FermDB on all time points measured (See accompanying manuscript by Bron *et al*. for correlation analyses details). The presence of 300 mM additional NaCl in the growth medium resulted in a significant (*P*<0.05) negative influence on *L. plantarum* GI-tract survival irrespective whether cells were analyzed after collection from either logarithmic or stationary phase of growth (shown for 60 min incubation in fig. 3A and B). *L. plantarum* grown in more acidic conditions (pH 5.2 instead of pH 6.4) and harvested in stationary phase showed a significantly (*P*<0.05) enhanced the gastric juice survival rate (shown for 60 min in fig. 3C).

### Transcriptome to Phenotype Association Identifies Candidate Effector Molecules for GI-tract Survival

In parallel with the GI-tract survival patterns, transcriptome profiles were obtained for logarithmic cells harvested from all fermentation conditions employed in this study (see accompanying paper by Bron *et al.*). To investigate whether high- and low-rate surviving cultures in the GI-tract assay could be distinguished based on the expression of specific genes, the cultures were first ranked on their GI-tract survival after gastric juice incubation (t = 60 min). For cultures that had retained undetectable survival rates after 60 min of gastric incubation, the relative survival rates after 20 min and 40 min of gastric incubation, were employed to refine their relative survival ranking (fig. 2A).

**Figure 4:**
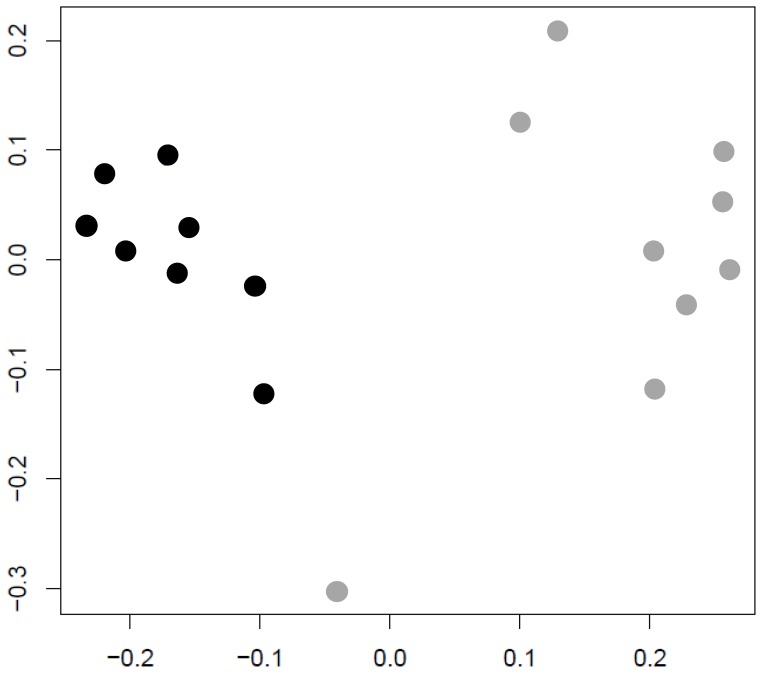
MDS plot of the eight best and the eight poorest surviving *L. plantarum* WCFS1 cultures grown under different growth conditions after GI-tract passage. Sample distances of good (black circles) and poor (grey circles) surviving cultures (see fig. 2A). Classification is based on the transcriptomes of these cultures just before subjection to the GI-tract survival assay.

The transcriptomes of the eight cultures with the highest survival rates and the eight cultures with the lowest survival in the GI-tract assay were clearly distinguishable according to principal component analysis (PCA) (fig. 4). This result indicated that the transcriptomes contained information (genes) within the first two components of the PCA which might allow the discrimination between high- and low-survival rates in the GI-tract assay. To identify specific transcripts that discriminate between low and high GI-tract survival, and thus can be regarded as candidate robustness markers, the random forest algorithm was applied (see the accompanying paper by Bron *et al*.). This allowed the identification of transcripts that have a high contribution to accurately predict the low- and high-survival outcomes (table S1).

### Validation of Target GI-tract Survival Effector Molecules by Mutagenesis

**Figure 5:**
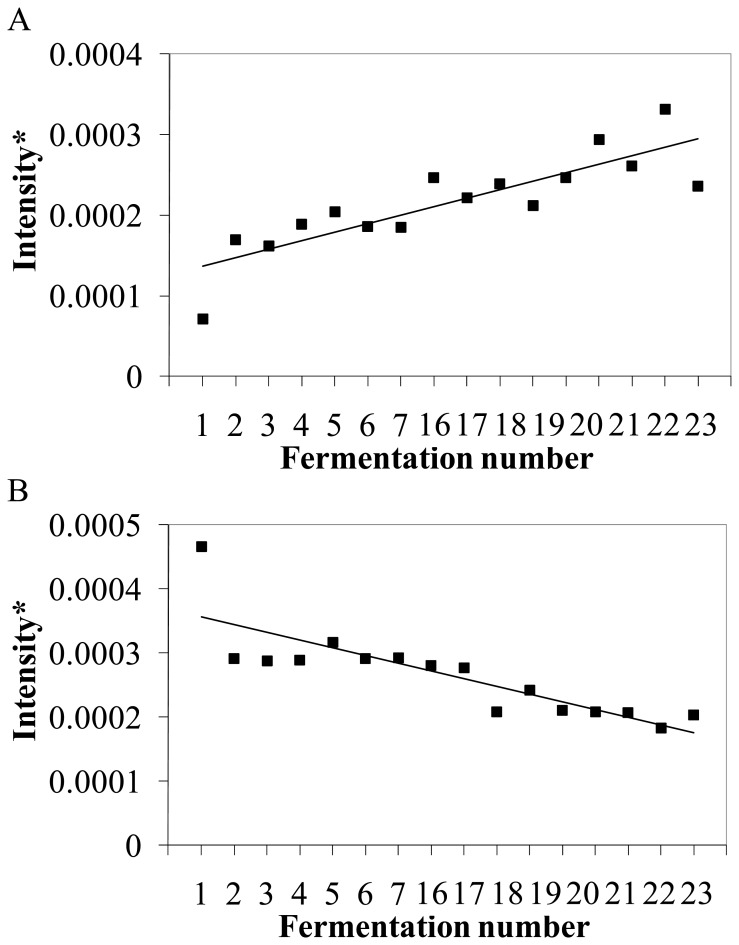
Correlation of *L. plantarum* WCFS1 GI-tract survival and transcript intensity of *thrC* (A) and *pbp2A* (B). The eight best and eight worst fermentations (see fig. 2A) are ranked with increasing GI-tract survival. *Data was normalized to correct for between slide variation [22]. R^2^ *thrC*  = 0.71, R^2^ *pbp2A*  = 0.70.

To validate the association of the expression level of specific genes in *L. plantarum* with GI-tract survival, the 13 genes with the highest ranking based on the criteria described in the Materials and Methods section were targeted by genetic engineering (table 1), aiming to improve GI-tract survival beyond the levels observed with the wild-type strain. Therefore, the direction of the correlation between transcript intensity and survival in the GI-tract assay determined whether a gene would be targeted for overexpression (positive correlation, see fig. 5A for an example) or gene-deletion (negative correlation, fig. 5B).

**Table 1:** Candidate genes linked with GI-tract survival of *L. plantarum* selected for genetic engineering.

ORF^a^	Name	function	Subcellular localization prediction^b^	Correlation withhigh survival^c^	R^2d^	Importance^e^	KO/over^f^	Strain^g^
lp_1413	*pbp2A*	transpeptidase-transglycosylase (penicillin binding protein 2A)	N-terminally anchored (No CS)	−	0.702	1.832	KO	NZ3412^CM^
lp_2827	*napA3*	Na(+)/H(+) antiporter	Multi-transmembrane	−	0.686	1.503	KO	NZ3416^CM^
lp_1669	*lp_1669*	transcription regulator, AraC family	Intracellular	−	0.601	1.156	KO	NZ3417^CM^
lp_3398	*pacL3*	cation transporting P-type ATPase	Multi-transmembrane	−	0.474	1.790	KO	NZ3415^CM^
lp_1817	*lp_1817*	ribitol-5-phosphate 2-dehydrogenase (putative)	Intracellular	−	0.378	1.156	KO	NZ3414^CM^
lp_2758	*thrC*	threonine synthase	Intracellular	+	0.714	1.227	over	pNZ3432^h^
lp_3299	*folB*	dihydroneopterin aldolase	Intracellular	+	0.638	1.772	over	pNZ7026^i^
lp_0149	*lp_0149*	ABC transporter, ATP-binding protein, Cobalt (orcobalamine)	Intracellular	+	0.634	1.977	over	pNZ3433^j^
lp_3297	*folE*	GTP cyclohydrolase I	Intracellular	+	0.554	1.356	over	pNZ7026^i^
lp_0148	*lp_0148*	ABC transporter, permease protein, Cobalt (or cobalamine)	Multi-transmembrane	+	0.523	1.156	over	pNZ3433^j^
lp_2349	*hicD3*	L-2-hydroxyisocaproate dehydrogenase	Intracellular	+	0.441	1.001	over	pNZ3431
lp_3296	*folC2*	folylpolyglutamate synthase/dihydrofolate synthase	Intracellular	+	0.432	1.081	over	pNZ7026^i^
lp_1357	*lp_1357*	extracellular protein, membrane-anchored (putative)	N-terminally anchored (No CS)	+	0.233	1.001	over	pNZ3430

a ORF, open reading frame.

b Subcellular localization prediction according to LocateP [54].

c +, positive correlation; -, negative correlation.

d R^2^ based on linear regression of transcript intensity and GI-tract survival of the eight best and eight worst surviving cultures (see fig. 2).

e Importance according to random forest [45].

f KO, knock out; over, overexpression.

g *L. plantarum* KO strains with NZ number or *L. plantarum* strains harboring plasmids (pNZ number).

h pNZ3432 contains *thrC* and *lp_2759*.

i pNZ7026 contains *folB*-*folK*-*folE*-*folC2*-*xtp2*-*folP*.

j pNZ3433 contains *lp_0148*, *lp_0149*, and *lp_0150*.

**Figure 6:**
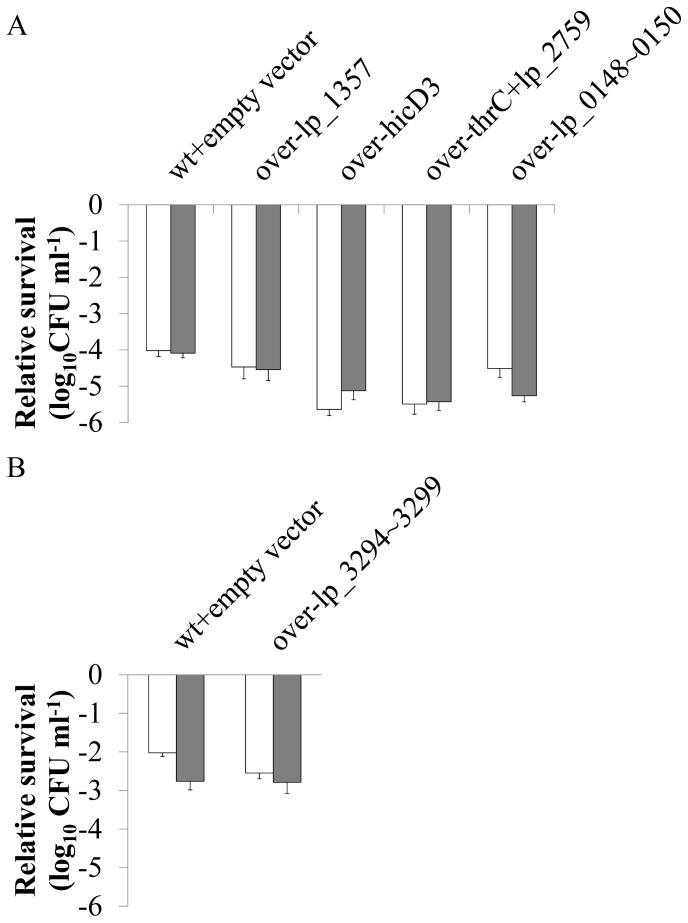
Relative GI-tract survival of *L. plantarum* mutants overexpressing genes potentially involved in GI-tract survival. Log_10_ CFU ml^−1^ determination of mid-exponentially grown in batch *L. plantarum* mutants after 60 min gastric juice incubation (white bars) and subsequent 60 min pancreatic juice incubation (grey bars). Input is set at 0 Log_10_ CFU ml^−1^. Empty vectors are pSIP411 (A) and pNZ7021 (B). *L. plantarum* harboring pNZ3430 (over*-lp_1357*), pNZ3431 (over*-hicD3*), pNZ3432 (over*-thrC*+*lp_2759*), pNZ3433 (over*-lp_0148∼0150*), and pNZ7026 (over*-lp_3294∼3299*). Data presented is the average of technical sextuplicates (+ standard deviation).

Genes targeted for sakacin-inducible overexpression were *thrC*, *lp_0149*, *hicD3*, and *lp_1357* (table 1). For *folB* overexpression, we used a previously constructed mutant that overexpresses the entire *folB*-*folK*-*folE*-*folC2*-*xtp2*-*folP* cluster [24], [25]. Sakacin P induced overexpression of the cytoplasmic *hicD3* and *thrC* and the downstream *lp_2759* gene products could readily be confirmed by SDS-PAGE analysis of cell-free extracts of induced cultures (figure S1). In contrast, overproduction of the membrane-anchored (*lp_1357*) and transmembrane proteins (*lp_0148–0150*) were not distinguishable by SDS-PAGE (data not shown). Although overexpression could only be demonstrated for two of the genetic loci, we applied all overexpression strains to our GI-tract assay. The constructed overexpression and gene deletion mutants were grown until the logarithmic growth phase and subjected to the GI-tract assay. The survival of the Sakacin P induced overexpression mutants was anticipated to improve when compared to a control strain harboring the empty induction plasmid (fig. 6). Although not significant, the contrary seemed to be the case, since the slight effects that were observed in some of the experiments suggested that the expression of the cloned genes reduced the survival capacity of these cells rather than improved.

**Figure 7:**
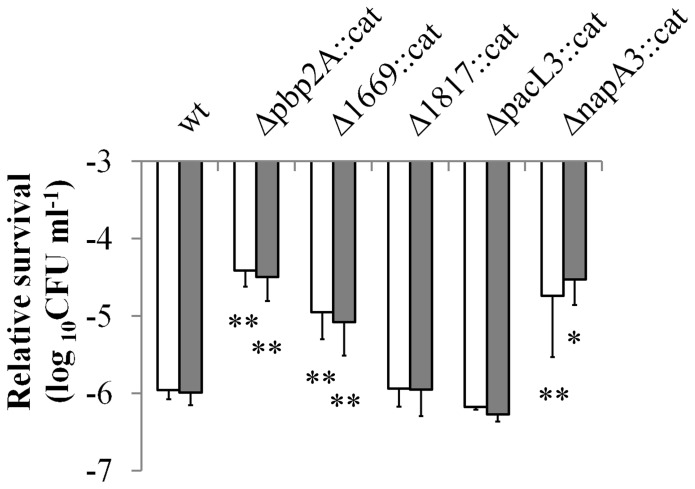
Relative GI-tract survival of *L. plantarum* mutants with *cat* replacements of candidate genes involved in GI-tract survival. Log_10_ CFU ml^−1^ determination of logarithmic (OD_600_ = 1.0) batch *L. plantarum* mutants after 60 min gastric juice incubation (white bars) and subsequent 60 min pancreatic juice incubation (grey bars). Input is set at 0 Log_10_ CFU ml^−1^. *Δpbp2A::cat*  =  *L. plantarum* NZ3412^CM^, *Δ1669::cat*  =  *L. plantarum* NZ3417^CM^, *Δ1817::cat*  =  *L. plantarum* NZ3414^CM^, *ΔpacL3::cat*  =  *L. plantarum* NZ3415^CM^, and *ΔnapA3::cat*  =  *L. plantarum* NZ3416^CM^. * *P-value* <0.05, ** *P-value* <0.01 compared with wild type (wt). Representative of two independent experiments, data presented are averages of technical sextuplicates (+ standard deviation).

In contrast, the *L. plantarum* Δ*pbp2A::cat*, Δ*lp_1669::cat*, and Δ*napA3::cat* mutants showed significantly improved survival in the GI-tract assay, as compared to their parental strain (fig. 7). These strains harbored disruptions in genes associated with poor survival in gastric stress. Notably, we have combined the individual mutants described here to construct Δ*pbp2A-*Δ*napA3::cat* and Δ*napA3-*Δ*lp_1669::cat*. However, these double gene deletion derivatives displayed robustness phenotypes comparable to the single Δ*napA3::cat* gene deletion derivative, indicating that the positive effect on GI robustness of these mutations appeared not cumulative (data not shown). Nevertheless, these results establish the involvement of certain fermentation-condition dependent gene products in GI survival.

Pbp2A is annotated as a penicillin-binding protein involved in peptidoglycan biosynthesis, Lp_1669 is predicted to be a transcription regulator, and NapA3 is homologous to Na^+^/H^+^ antiporters. To gain more insight in the mechanisms by which these proteins influence robustness, growth of the parental strain and the Δ*pbp2A::cat*, Δ*lp_1669::cat*, and Δ*napA3::cat* derivatives was monitored under standard- and stress-conditions. At 28°C in complex culture medium (MRS), the growth rates of the mutants did not differ from the wild-type, nor did the addition of H_2_O_2_ (1 to 5 mM), lysozyme (0.025 to 3.2 g/ml), or SDS (0.9 to 30 g/l) induce differences in growth rate of the mutants compared with the wild type strain. However, the presence of bile salts (10 to 50 mM) in the culture medium reduced the maximum growth rate of Δ*pbp2A::cat* to 20% as compared to the parental strain (data not shown). This result indicates that Pbp2A contributes to the survival capacity of *L. plantarum* in low-pH, stomach like conditions, but also improves bile tolerance, but not to tolerance to detergents in general.

The addition of NaCl to the growth medium reduced the growth rate of *ΔnapA3::cat* to 20% (400 mM) and 80% (1 M) of the wild type (data not shown). As NapA3 is a Na^+^/H^+^ antiporter which might be affected by extracellular pH, the growth of the Δ*napA3::cat* mutant was monitored under different starting pH conditions (pH 4.6 to 6.4) in the presence and absence of NaCl (300 mM). The growth rate of the mutant appeared unaltered during growth in the absence of salt. Only the presence of NaCl reduced the growth rate of *ΔnapA3::cat* under all measured conditions (data not shown). These results support a role of this function in salt tolerance, which in our experiments, appeared to be independent of the pH.

**Figure 8:**
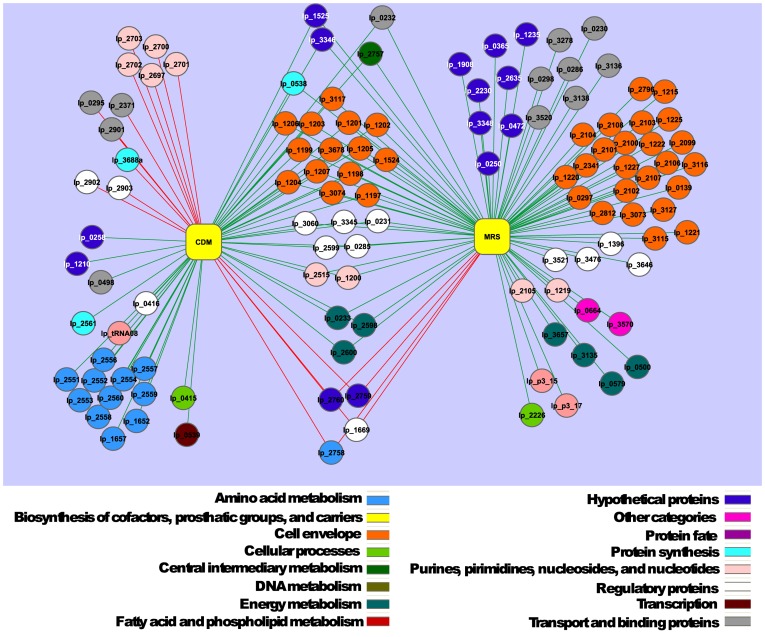
Regulatory network of Lp_1669 grown in 2× CDM or MRS. Yellow rectangle nodes represent growth in 2× CDM (left node) or in MRS (right node). Round nodes represent single genes with their corresponding lp_number, which is the number as annotated in the sequenced parental *L. plantarum* WCFS1 strain [16], green lines represent down-regulation, and red lines represent up-regulation of the gene compared to the parental strain. The colors of the round nodes represent the annotated main class. See figure S2 of the supplemented materials for the original cytoscape file.

Contrary to Δ*napA3::cat* and Δ*pbp2A::cat*, a specific phenotype was not established for the transcription regulator Lp_1669. To elucidate the regulon associated with this regulator, the transcriptome profile of the NZ3417^CM^ (Δ*lp_1669*::*cat*) strain was compared to that of the wild-type strain grown in 2× CDM [17] or MRS. The results showed that the Lp_1669-deficient strain displayed enhanced expression of genes belonging to the main functional class of cell envelope associated functions, and more specifically to its subclass of surface polysaccharides, lipopolysaccharides, and antigens. This effect of the mutation was observed independent of the medium used (fig. 8 and table S2 and S3). Analysis at the individual transcript level revealed that the capsular polysaccharide (CPS) clusters *cps2*, *cps3*, and *cps4* were induced in the MRS-grown Lp_1669-deficient strain as compared to the wildtype, suggesting that the regulatory function encoded by *lp_1669* is involved, either directly or indirectly, in the regulation of CPS biosynthesis. Notably, especially the expression of the *cps2* cluster was induced in 2× CDM grown Lp1669 deficient cells (table S2 and S3). Analysis of monosaccharide composition revealed minor changes in CPS sugar composition of the Lp_1669-deficient strain in comparison to the wild type strain (table 2). Galactosamine was only detected in the mutant strain, whereas arabinose was found only in the wild-type strain. Rhamnose and glucosamine also tended to be slightly more abundant in the wild type *L. plantarum* WCFS1. Moreover, the average molar mass of Δ*lp_1669::cat* strain-derived CPS was 1.5-fold higher compared to the wild type (Table 2). This indicates that Lp_1669 seems to be involved in subtle CPS modification, specifically in chain length determination. These observations might also (partially) explain the observed increased gastrointestinal survival of the *L. plantarum* Lp_l669-deficient strain.

**Table 2:** Molar mass and sugar composition of CPS isolated from *L. plantarum* WCFS1 and NZ3417^CM^ (*Δlp_1669::cat*).

Strain	WCFS1	*Δlp_1669::cat*
Total molar mass (kg/mol)	20 (±1.4)	30 (±1.5)
**Sugar (% of total sugars)** a		
Rhamnose	3.2	2.6
Galactosamine	ND	1.3
Arabinose	0.5	ND
Glucosamine	3.7	2.8
Galactose	12.6	12.8
Glucose	27.8	26.4
Galacturonic acid	52.3	54.1

a ND is not detected.

## Discussion

This study demonstrates that the production method, medium composition, and stage of growth strongly influenced the GI-survival efficacy of *L. plantarum* WCFS1. Combining the fermentation and survival data pinpointed to specific fermentation conditions that may enhance robustness (low salt and low pH), whereas genome association analysis of the transcriptome and survival data revealed 13 genes potentially involved in GI-survival.

As reported previously [26], cells harvested from stationary phase generally were more robust than logarithmically growing cells, and in particular, those cells displayed enhanced survival in gastric juice which overall had a dramatically larger impact on survival compared to pancreatic juice. The influence of acidity on GI-tract survival was also emphasized by the observation that lowering the gastric juice pH by as little as 0.1 unit had a pronounced impact on survival. Differences among bacterial species in their sensitivity to gastric and intestinal secretions have been observed before [27], [28], [29] and a higher sensitivity for acid than bile stress was also noted for *L. rhamnosus*, as well as for other *L. plantarum* strains [30], [31]. The finding that exposure to low pH during growth enhances GI-survival is in agreement with earlier observations that pre-adaptation to sublethal stress conditions enhances the subsequent robustness of bacteria to lethal stress conditions [9], supporting the suitability of the fermentation genomics platform and bioinformatics tools employed in this study and the accompanying paper by Bron *et al*. For salt it is known that it can protect against, but also increase susceptibility to, other stresses [32], [33]. Moreover, these results clearly establish that fermentation conditions have a major impact on the GI-tract associated stress tolerance of bacterial cultures, and that specifically mild salt stress and lower pH adaptation may elicit adaptive responses that reduce and support such stress tolerance, respectively.

Overexpression and gene deletion derivatives of the parental strain were constructed, depending on the direction of the predicted correlation to GI-tract survival and aiming to enhance this trait. Three of the five constructed gene deletion derivatives displayed enhanced GI-tract survival, confirming the predicted role of the targeted genes. By contrast, none of the overexpression derivatives displayed improved robustness behavior in the GI-tract assay, and all had survival characteristics that were virtually identical to those of the parental strain. A possible explanation for these observations may be found in the potential disruption of a gene-regulatory network by the deletion of a single gene in that network, while overexpression of a single element from a complementary gene-function network may not provide the same effect as the enhanced expression of all elements in the network. The fact that 3 out of 5 gene deletion derivatives displayed enhanced survival is in line with earlier observations [21], [22], [23] and can be explained by the fact that the random forest algorithm also leads to the identification of non-causal relationships, reiterating the importance of follow-up mutagenesis approaches to establish a definite role for candidate biomarkers identified with this algorithm.

The 3 genes for which the importance in GI-tract survival could be confirmed by gene deletion encode a AraC family regulator (Lp_1669), a Na^+^/H^+^ antiporter; NapA3, and a penicillin binding protein; Pbp2A. Notably, all three proteins are associated with cell wall modification and transport, and their mutation may lead to cell envelope modulation. This finding per se, may not be qualified as surprising, because the cell envelope is the first line of defense against stresses [34]. Moreover, the resistance to acid and adaptation to bile stress in *L. plantarum* WCFS1 has been associated with membrane integrity and cell envelope modifications, respectively [35], [36]. The AraC family of regulators to which Lp_1669 belongs [16] is characterized by transcriptional regulators that act mostly as activators. However, in some cases these regulators serve as repressors of transcription or as both activators and repressors [37]. The observed effect of Lp_1669 on GI-tract survival is likely to be indirect, possibly via CPS remodeling, because the Lp_1669-deficient strain had CPS with a higher molar mass that might result in a thicker CPS layer around the cells. It has been demonstrated that the presence of EPS/CPS improved the *in vivo* GI survival of *L. rhamnosus* GG [38]. The Na^+^/H^+^ antiporter NapA3 might affect GI survival via a role in pH homeostasis. Because disruption of *napA3* improved GI-tract survival, it seems likely that NapA3 exports sodium ions associated with the influx of protons, thereby decreasing its internal pH and proton motive force due to the acid stomach conditions. This is also in line with our observation that the gene deletion derivative is only reduced during growth in the presence of sodium salts. Finally, *pbp2A* encodes the penicillin binding protein 2A which is annotated to be involved in peptidoglycan biosynthesis [16]. Disruption of *pbp2A* improved the acid stomach condition survival, while it decreased the growth rate in the presence of bile. Noteworthy in this respect is the finding that the compositions of peptidoglycan directly affects the integrity of the cells and can influence the acid- and bile-tolerance [9], [39], [40], [41]. Moreover, transcriptome analysis of *L. acidophilus* NCFM and *L. plantarum* WCFS1 demonstrated that many genes related to cell membrane and peptidoglycan biosynthesis displayed altered expression profiles during exposure to bile [39], [42]. An increased acid sensitivity by the inactivation of penicillin binding proteins is found in *Lactococcus lactis* and *L. reuteri* [9], [40]. However, we found the deletion of *pbp2A* improves the GI-tract survival, which suggests that disruptions in peptidoglycan biosynthesis genes could either improve or decrease the survival of probiotics, reiterating the general concept of subtle inter-strain and species differences in survival mechanisms.

In conclusion, this study demonstrated that fermentation conditions have a large influence on the GI-tract survival of *L. plantarum*. We showed that transcriptome-trait matching enables the identification of genetic loci involved in gastrointestinal robustness and this approach can also be employed to rationally design fermentation and process conditions that aim for the production of probiotics with improved GI survival and consequently have a higher potential to achieve their desired health-beneficial effects on the consumer.

## Materials and Methods

### GI-tract Assay and Correlation to Transcriptome Data

Cells were harvested from the fermentation-genomics platform at OD_600_ = 1.0 for full-genome transcriptome profiling (see accompanying paper by Bron *et* al.), while the GI-tract survival was determined in the same cells, as well as in cells that were harvested 25 h after inoculation. One set of fermentations (F19–F24, see accompanying paper by Bron *et al.*) was excluded from the data analysis as the GI-tract survival data appeared unreliable, likely caused by minor deviations in the pH of the batch of GJ applied which is known to heavily influence GI survival. For GI-tract survival analysis, cultures were washed with prewarmed (37°C) PBS and resuspended in prewarmed (37°C) filter sterilized gastric juice [53 mM NaCl, 15 mM KCl, 5 mM Na_2_CO_3_, 1 mM CaCl_2_, 0.1 mg ml^−1^ lipase (Fluka; derived from *Aspergillus niger*), and 1.2 mg ml^−1^ pepsin (Sigma; derived from porcine) that had a pH adjusted to 2.4 with HCl (logarithmic cells) or 2.3 (stationary cells)]. The gastric juice enzymes were added immediately prior to the treatment. After 60 min incubation while rotating at 10 rpm in a Hybridization oven/shaker (Amersham pharmacia biotech, Little Chalfont, UK) at 37°C, the cultures were neutralized to pH 6.5 with 0.5 M NaHCO_3_, and prewarmed (37°C) pancreatic juice [85 mM NaCl, 5 mM KH_2_PO_4_, 2 mM Na_2_HPO_4_, 10 mM NaHCO_3_, 30 mg ml^−1^ pancreatin (Sigma; derived from porcine stomach) and bile acid mixture (latter two components were added fresh to pancreatic juice immediately prior to the treatment)] was added, followed by continued incubation for another 60 min (agitation at 10 rpm, 37°C). The bile acid mixture consisted of 3.0 mM (final concentration in assay) sodium glycocholate hydrate, 1.3 mM sodium glycodeoxycholate, 2.4 mM sodium glycochenodeoxycholate, 1.0 mM taurocholic acid sodium salt hydrate, 0.4 mM sodium taurodeoxycholate hydrate and 1.0 mM sodium taurochenodeoxycholate to mimic human bile components and concentrations [43]. Preceding and during GI-tract assay incubation (t = 0, 20, 40, 60, 90, and 120), samples were taken for colony forming unit (CFU) enumeration by spot-plating [44]. A reduction of 8 logs could be detected with this method. Relative GI-tract survival of the different cultures was expressed as the fraction of the corresponding input numbers of viable cells (t = 0 was set at 1.00). The transcriptome and GI-tract survival data are available in FermDB (www.cmbi.ru.nl/fermdb).

The initial list of genes predicted by the random forest algorithm [45] (see the accompanying paper by Bron *et al*. for details on correlation analyses) to be associated with GI-tract survival was further refined by application of several selection criteria that are based on transcript ranking. Firstly, only transcripts with an importance factor higher than 1 according to the random forest algorithm were selected for further analysis. Secondly, the quantitative correlation of individual transcripts with the survival rate observed in individual cultures was evaluated, selecting those transcripts (genes) that had the highest quantitative correlation with survival (expressed in R^2^ in table S1, see figure 5 for two examples). Lastly, genes encoding prophage associated functions that are typically hypervariable among *L. plantarum* strains were discarded [13], [20]. The remaining transcripts and their associated genes (table 1) were considered to have the strongest correlation with the measured gastric juice tolerance and were therefore selected for validation by gene deletion or overexpression.

### Deletion Mutant Construction

Gene deletion mutants were constructed using the mutagenesis vector pNZ5319 according to Lambert et al. [19]. The *L. plantarum* WCFS1 *pbp2A, lp_1669, lp_1818, pacL3,* and *napA3* genes were replaced with a *lox66*-P_32_-*cat-lox71* cassette resulting in strains NZ3412^CM^ (Δ*pbp2A::cat*), NZ3417^CM^ (Δ*lp_1669::cat*), NZ3414^CM^ (Δ*lp_1817::cat*), NZ3415^CM^ (Δ*pacL::cat*), and NZ3416^CM^ (Δ*napA3::cat*), respectively. Primer sequences used to construct the gene-targeted knock-out vectors for *L. plantarum* WCFS1 are provided in table S4. In short, upstream and downstream flanking regions (left flank, LF; right flank, RF, respectively) of the target genes (i.e., *pbp2A, lp_1669, lp_1817, pacL3,* and *napA3*) were amplified with primer pair combinations as listed in table S5. Primers at the 3′-end of the upstream and 5′-end of the downstream flanking regions (A3, A4, B3, B4, C3, C4, D3, D4, E3, and E4 ) were extended with an overlap-sequence complementary to the 5′ and 3′ end of the *lox66*-P_32_-*cat-lox71* cassette (amplified with primers I and J [46]), to enable knock-out construction by a Splicing by overlap extension (SOE) PCR [47] with primer pairs as listed in table S5. The obtained (SOE-ing) amplicons were blunt-ligated into *Ecl*136II-*Swa*I digested pNZ5319 [19] resulting in plasmids pNZ3412, pNZ3417, pNZ3414, pNZ3415, and pNZ3416 (see table 3). *Escherichia coli* was used as an intermediate cloning host and after introduction of the mutagenesis plasmids into competent *L. plantarum* WCFS1, cells were plated on MRS containing 10 µg ml^−1^ chloramphenicol. After 48 h, grown colonies were plated on MRS with and without 30 µg ml^−1^ erythromycin. Colonies from each mutant displaying the anticipated erythromycin sensitive phenotype were selected for colony-PCR using primer pairs as listed in table S5. Mutant colonies with the expected genetic organization were selected for each of the knock-out target loci; NZ3412^CM^ (Δ*pbp2A::cat*), NZ3417^CM^ (Δ*lp_1669::cat*), NZ3414^CM^ (Δ*lp_1817::cat*), NZ3415^CM^ (Δ*pacL::cat*) and NZ3416^CM^ (Δ*napA3::cat*). The *L. plantarum* WCFS1 *pbp2A* plus *napA3* and *napA3* plus *lp_1669* double-mutants were constructed in the NZ3412^CM^ (Δ*pbp2A::cat*) and NZ3416^CM^ (Δ*napA3::cat*) background, respectively, in a two-step procedure. Firstly, strains NZ3412 (Δ*pbp2A*) and NZ3416 (Δ*napA3*) were constructed by excision of the *lox66*-P_32_-*cat-lox71* cassette by transient expression of the Cre resolvase enzyme from pNZ5348 according to methods described by Lambert et al. [19]. In these deletion mutant strains, pNZ3416 and pNZ3417 were introduced and double mutant strains were selected using the approach described above, resulting in the isolation of strains NZ3419^CM^ (Δ*pbp2A-*Δ*napA3::cat*) and NZ3418^CM^ (Δ*napA3-*Δ*lp_1669::cat*), respectively (table 3).

**Table 3:** Strains and plasmids used in this study.

Strain or plasmid	Relevant feature(s)^a^	Reference
**Strains**		
*L. plantarum*		
WCFS1	Single-colony isolate of *L. plantarum* NCIMB8826	[16]
NZ3412^CM^	Derivative of WCFS1 containing a *lox66*-P32-cat-*lox71* replacement of *pbp2A* (Δ*pbp2A::cat*)	This work
NZ3412	Derivative of WCFS1 containing a *lox72* replacement of *pbp2A* (Δ*pbp2A*)	This work
NZ3417^CM^	Derivative of WCFS1 containing a *lox66*-P32-cat-*lox71* replacement of *lp_1669* (Δ*lp_1669::cat*)	This work
NZ3414^CM^	Derivative of WCFS1 containing a *lox66*-P32-cat-*lox71* replacement of *lp_1817* (Δ *lp_1817::cat*)	This work
NZ3415^CM^	Derivative of WCFS1 containing a *lox66*-P32-cat-*lox71* replacement of *pacL3* (Δ *pacL3::cat*)	This work
NZ3416^CM^	Derivative of WCFS1 containing a *lox66*-P32-cat-*lox71* replacement of *napA3* (Δ*napA3::cat*)	This work
NZ3416	Derivative of WCFS1 containing a *lox72* replacement of *napA3* (Δ*napA3*)	This work
NZ3419^CM^	Derivative of NZ3412 containing a *lox66*-P32-cat-*lox71* replacement of *napA3* (Δ*pbp2A*-Δ*napA3::cat*)	This work
NZ3418^CM^	Derivative of NZ3416 containing a *lox66*-P32-cat-*lox71* replacement of *napA3* (*ΔnapA3*-Δ*lp_1669::cat*)	This work
SIP411	Derivative of WCFS1 harboring the pSIP411 plasmid	This work
SIP411B	Derivative of WCFS1 harboring the pSIP411B plasmid (empty vector)	This work
NZ3430	Derivative of WCFS1 harboring the pNZ3430 plasmid (over-*lp_1357*)	This work
NZ3431	Derivative of WCFS1 harboring the pNZ3431 plasmid (over-*hicD3*)	This work
NZ3432	Derivative of WCFS1 harboring the pNZ3432 plasmid (over-*thrC* and *lp_2759*)	This work
NZ3433	Derivative of WCFS1 harboring the pNZ3433 plasmid (over-*lp_0148∼0150*)	This work
NZ7021	Derivative of WCFS1 harboring the pNZ2021 plasmid (empty vector)	[24]
NZ7026	Derivative of WCFS1 harboring the pNZ2026 plasmid (over-*folB, folP, folk, folE, xtp2,* and *folC2*)	[24]
*E. coli*		
TOP-10	Cloning host; F- *mcrA* Δ(*mrr*-*hsdRMS*-*mcrBC*) φ80*lacZ*Δ*M15* Δ*lacX74 nupG recA1 araD139* Δ(*ara-leu*)*7697 galE15 galK16 rpsL*(Str^r^) *endA1* λ^-^	Invitrogen
MC1061	Cloning host; *araD139* Δ(*araA-leu*)7697 Δl*acX74 galK16 galE15*(*GalS*) λ^-^ *e14^-^ mcrA0 relA1 rpsL150*(str^r^)*spoT1 mcrB1 hsdR2*	[55]
**Plasmids**		
pNZ5319	Cm^r^ Em^r^; for multiple gene replacements in Gram-positive bacteria	[19]
pNZ3412	Cm^r^ Em^r^; pNZ5319 derivative containing homologous regions up- and downstream of WCFS1 *pbp2A*	This work
pNZ3417	Cm^r^ Em^r^; pNZ5319 derivative containing homologous regions up- and downstream of WCFS1 *lp_1669*	This work
pNZ3414	Cm^r^ Em^r^; pNZ5319 derivative containing homologous regions up- and downstream of WCFS1 *lp_1817*	This work
pNZ3415	Cm^r^ Em^r^; pNZ5319 derivative containing homologous regions up- and downstream of WCFS1 *pacL3*	This work
pNZ3416	Cm^r^ Em^r^; pNZ5319 derivative containing homologous regions up- and downstream of WCFS1 *napA3*	This work
pSIP411	Em^r^; cloning vector	[48]
pSIP411B	Em^r^; pSIP11 derivative without the *gusA* gene (empty vector)	This work
pNZ3430	Em^r^; pSIP411 derivative containing the *lp_1357* gene of WCFS1	This work
pNZ3431	Em^r^; pSIP411 derivative containing the *hicD3* gene of WCFS1	This work
pNZ3432	Em^r^; pSIP411 derivative containing the *thrC* and *lp_2759* operon of WCFS1	This work
pNZ3433	Em^r^; pSIP411 derivative containing the *lp_0148∼0150* operon of WCFS1	This work
pNZ7021	Cm^r^; (empty vector)	[24]
pNZ7026	Cm^r^; pNZ7021 derivative containing the *folB, folP, folk, folE, xtp2,* and *folC2* gene cluster of WCFS1	[24]
pNZ5348	Em^r^; containing *cre* under the control of the *pcrA* (*lp_1144*) promoter	[19]

a Str^r^, streptomycin resistant; Cm^r^ chloramphenicol resistant; Em^r^, erythromycin resistant.

### Overexpression Mutant Construction and SDS-PAGE Analysis

Gene overexpression mutants were constructed using the expression vector pSIP411 [48]. For the candidate genes selected for overexpression that were part of a predicted operon [49], the whole operon was cloned in the sakacin induction vector (table 1). Primers were designed (table S5) to introduce a restriction enzyme site for cloning the target gene(s) into the expression vector pSIP411 at the *Nco*I site. The *lp_1357* and *thrC+lp_2759* overexpression mutants were designed with *BspH*I site, which has compatible ends with *Nco*I site. The target gene(s) were amplified by PCR using corresponding primers for each mutant (F1/F2, G1/G2, H1/H2 and I1/I2 for *lp_1357, lp_2349, thrC+lp_2759*, and *lp_0148∼0150* mutants, respectively). The reactions were carried out with KOD polymerase (Novagen, Darmstadt, Germany) according to the instructions of the manufacturer. The purified PCR products were digested by restriction enzymes (Invitrogen, Molecular probes, Inc, USA) for which sites were introduced in the primers (see table S4) and cloned in *Nco*I-*Sma*I digested pSIP411. Ligation mixtures were transformed to *E. coli*, and re-isolated from primary transformants. Correctly assembled overexpression plasmids were identified by PCR, restriction and sequence analysis. Re-isolated plasmids were propagated into *L. plantarum* WCFS1 and transformants were selected on MRS containing 30 µg·ml^−1^ erythromycin (table 3).

For protein analysis of the overexpression mutants, the induction and sample preparation procedures were modified from the description by Sørvig et al. [48]. The 19-amino-acid inducing peptide (of Met-Ala-Gly-Asn-Ser-Ser-Asn-Phe-Ile-His-Lys-Ile-Lys-Gln-Ile-Phe-Thr-His-Arg [50]) was custom-synthesized by BACHEM (Budendorf, Switzerland). The inducing peptide was dissolved in degassed water, as recommended by BACHEM to avoid oxidation of the peptides. The overnight cultures of the overexpression strains were diluted 50-fold and then incubated at 37°C. After OD_600_ had reached 0.3, the inducing peptide was added to the cultures at varying concentrations of 0, 0.1, 1, 10, and 50 ng/ml. Incubation was continued at 37°C for another 4 h until the OD_600_ had reached approximately 1.8. Bacterial cells were collected by centrifugation at 5,200×*g* for 10 min, followed by resuspension of the cell pellet in 50 mM Sodium-phosphate buffer pH 7. The cells were disrupted with 1 g zirconium beads by using a FastPrep™ (Qbiogene Inc, Cedex, France). After the disruption, the samples were centrifuged 5 min at 20,800×*g* to obtain cell-free extracts for analysis by SDS-PAGE.

### DNA Microarray Analysis and Data Visualization

DNA microarray analysis were performed to compare global transcriptome profiles of NZ3417^CM^ (Δ*lp_1669::cat*) and the wild-type. RNA isolation from *L. plantarum*, subsequent cDNA synthesis and indirect labeling, as well as DNA microarray hybridizations were performed as described in the accompanying paper by Bron *et al*. The hybridization scheme is presented in figure S3. Genes of the Lp_1669 regulon with FDR-adjusted p-values less than 0.05 together with a fold-change higher than 2.0 or lower than 0.5 were considered to be significantly differently expressed. All microarray data is MIAME compliant and is available in the GEO database under accession number GSE31254. The biomolecular interaction network of the Lp_1669 regulon in 2× CDM and MRS was visualised using the Cytoscape software (version 2.8.1) [51], and the Biological Networks Gene Ontology (BiNGO) tool [52] was employed to detect significantly overrepresented categories in the regulon of Lp_1669. See the accompanying paper by Bron *et al*. for details.

### Phenotypic Assays of Mutant Strains

Gene deletion mutants were analyzed for their gastrointestinal survival characteristics in a procedure identical to that described for the wild-type (see above). To evaluate the relative GI-tract survival of the overexpression mutants, the mutant strain SIP411B (empty vector) and the overexpression mutants were sakacin-induced (50 ng/ml) (see above). Additionally, to measure the relative GI-tract survival of the folate overexpression strain, strains NZ7021 (empty vector) and NZ7026 (folate overproducing strain) [25] were inoculated at OD_600_ = 0.1 in MRS containing 80 mg/ml chloramphenicol and 0 or 10 mg/ml *p*-aminobenzoic acid (*p*ABA) according to Wegkamp et al. [24], grown at 37°C until OD_600_ was 1.0, and subjected to the GI-tract survival assay. To evaluate relative growth efficiency of the deletion mutants, the parental strain (WCFS1) and mutant strains NZ3412^CM^ (Δ*pbp2A::cat*), NZ3417^CM^ (Δ*lp_1669::cat*), and NZ3416^CM^ (Δ*napA3::cat*) were inoculated at OD_600_ = 0.1 in 96-wells plates and incubated in MRS broth at 28°C. OD_600_ of the cultures was monitored spectophotometrically (GENios, Tecan Austria GmbH, Grödig, Austria).

### Capsular Polysaccharide Isolation and Determination

Capsular polysaccharide (CPS) was purified and chain lengths and sugar composition were determined essentially as described before [53]. Briefly, 500 ml cultures of *L. plantarum* WCFS1 and NZ3417^CM^ (Δ*lp_1669::cat*) were grown in 2× CDM at 37°C until stationary phase (25 h). After 1 h incubation at 55°C, the cells were separated from the CPS containing growth medium by centrifugation for 15 min (6000×*g*) and to prevent overgrowth during dialysis, erythromicine was added to the supernatant to a final concentration of 10 µg/ml. A dialyzing tube 12–1400 Da (Fisher Scientific) was prepared by boiling twice 2% NaHCO_3_/2 mM EDTA, and once in reverse osmosis water. After overnight dialysis against running tap water followed by 4 h dialysis using reverse osmosis water, the samples were freeze-dried and stored at −20°C until further analysis.

The samples were dissolved in eluent (100 mM NaNO_3_+0.02% NaN_3_), filtered over 0.2 µm, and placed in a thermally controlled sample holder at 10°C and 200 µl was injected (model 231 Bio, Gilson) on the columns connected in series and remained at 35°C with a temperature control module (Waters, Milford, USA) to perform size exclusion chromatography (SEC) [TSK gel PWXL guard column, 6.0 mm×4.0 cm, TSK gel G6000 PWXL analytical column, 7.8 mm×30 cm, 13.0 µm and TSK gel G5000 PWXL analytical column, 7.8 mm×30 cm, 10 µm (TosoHaas, King of Prussio, USA)]. Light scattering was measured at 632.8 nm at 15 angles between 32° and 144° (DAWN DSP-F, Wyatt Technologies, Santa Barbara, USA). UV absorption was measured at 280 nm (CD-1595, Jasco, de Meern, The Netherlands) to detect proteins. The specific viscosity was measured with a viscosity detector (ViscoStar, Wyatt Technologies, Santa Barbara, USA) at 35°C and sample concentration was measured by refractive index detection, held at a fixed temperature of 35°C (ERC-7510, Erma Optical Works, Tokyo, Japan).

During the analysis with SEC the polysaccharide peak was collected (2 min×0.5 mL/min  = 1 mL). The acid hydrolyses of the collected polysaccharide was carried out for 75 min at 120°C with 2 M trifluoro acetic acid under nitrogen. Following hydrolyses, the solutions were dried overnight under vacuum and dissolved in water. High Performance Anion Exchange Chromatography with Pulsed Amperometric Detection (HPAEC-PAD) on a gold electrode was used for the quantitative analyses of the monosaccharides rhamnose, galactosamine, arabinose, glucosamine, galactose, glucose, mannose, xylose, galacturonic acid, and glucuronic acid. The analyses were performed with a 600E System controller pump (Waters, Milford, USA) with a helium degassing unit and a model 400 EC detector (EG&G, Albuquerque, USA). With a 717 autosampler (Waters, Milford, USA), 20 µl of the sample was injected on a Dionex Carbopac PA-1, 250×4 mm (10–32), column thermostated at 30°C. The monosaccharides were eluted at a flow rate of 1.0 mL/min. The monosaccharides were eluted isocratic with 16 mM sodium hydroxide, followed by the elution of the acid monosaccharides starting at 20 min with a linear gradient to 200 mM sodium hydroxide +500 mM sodium acetate in 20 minutes. Data analysis was done with Dionex Chromeleon software version 6.80. Quantitative analyses were carried out using standard solutions of the monosaccharides (Sigma-Aldrich, St. Louis, USA).

## Supporting Information

Figure S1 **SDS-PAGE of cell-free extracts logarithmic** ***L. plantarum*** **strains overexpressing** ***hicD3*** **(** ***lp_2349*** **) and overexpressing** ***thrC*** **(** ***lp_2758*** **) and** ***lp_2759*** **.** The arrows indicate protein bands increasing with increasing amounts of Sakacin P (inducing peptide, IP). Empty vector  =  pSIP411B. *L. plantarum* harboring pNZ3431 (over*-hicD3*), and pNZ3432 (over*-thrC*+*lp_2759*). Marker sizes are indicated in kDalton (kDa).
(TIF)

Figure S2 **Cytoscape version of** **figure 8** **; regulatory network of Lp_1669 grown in 2× CDM or MRS.** Yellow rectangle nodes represent growth in 2× CDM (left node) or in MRS (right node). Round nodes represent single genes with their corresponding lp_number, which is the number as annotated in the sequenced parental *L. plantarum* WCFS1 strain [16], green lines represent down-regulation, and red lines represent up-regulation of the gene compared to the parental strain. The colors of the round nodes represent the annotated main class.
(CYS)

Figure S3 **Lp_1669 regulon hybridization scheme.** Tail and head of the arrow represent Cy3 and Cy5 labeling, respectively.
(TIF)

Table S1 **Candidate genes associated with GI-tract survival of** ***L. plantarum*** **WCFS1.**
(DOCX)

Table S2 **Differentially regulated genes in NZ3417^CM^ (Δ** ***lp_1669::cat*** **) grown in 2× CDM.**
(DOCX)

Table S3 **Differentially regulated genes in NZ3417^CM^ (Δ** ***lp_1669::cat*** **) grown in MRS.**
(DOCX)

Table S4 **Primers used in this study.**
(DOCX)

Table S5 **Primer pair combinations used for LF and RF amplification and for the SOE step of the deletion mutants.**
(DOCX)

Table S6 **Primer pair combinations used for each deletion mutant to confirm the correct integration in the genome.**
(DOCX)
