# Supplementary figures and images for: Modulation of Lactobacillus plantarum Gastrointestinal Robustness by Fermentation Conditions Enables Identification of Bacterial Robustness Markers
Source: PLoS One. 2012 Jul 3;7(7):e39053. doi: 10.1371/journal.pone.0039053 (PMC3389004; doi:10.1371/journal.pone.0039053)

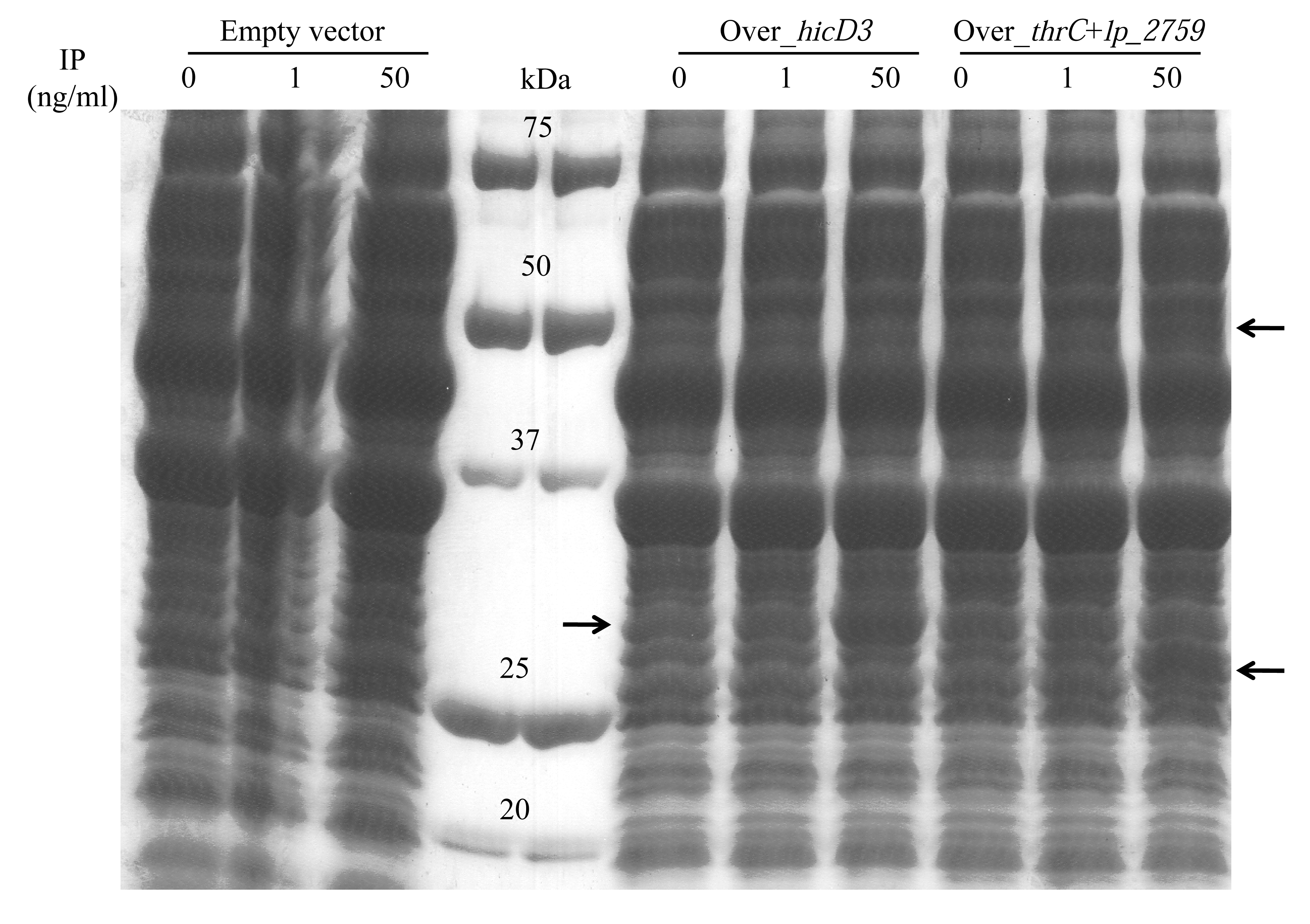

Supplement: Figure S1 — SDS-PAGE of cell-free extracts logarithmic L. plantarum strains overexpressing hicD3 ( lp_2349 ) and overexpressing thrC ( lp_2758 ) and lp_2759 . The arrows indicate protein bands increasing with increasing amounts of Sakacin P (inducing peptide, IP). Empty vector = pSIP411B. L. plantarum harboring pNZ3431 (over-hicD3), and pNZ3432 (over-thrC+lp_2759). Marker sizes are indicated in kDalton (kDa). (TIF) [file pone.0039053.s001.tif]

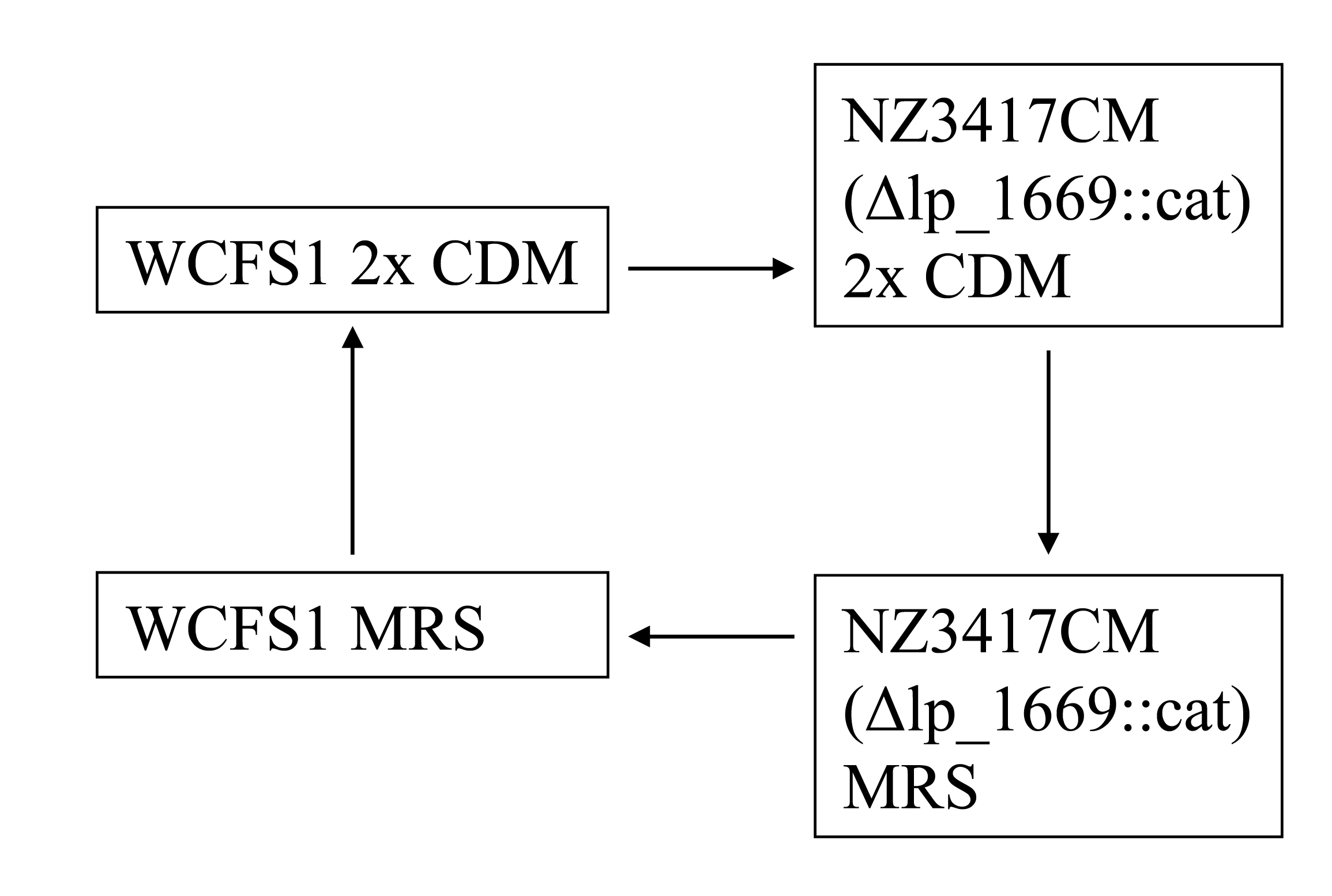

Supplement: Figure S3 — Lp_1669 regulon hybridization scheme. Tail and head of the arrow represent Cy3 and Cy5 labeling, respectively. (TIF) [file pone.0039053.s003.tif]
