# Supplementary material for: Modulation of Lactobacillus plantarum Gastrointestinal Robustness by Fermentation Conditions Enables Identification of Bacterial Robustness Markers
Source: PLoS One. 2012 Jul 3;7(7):e39053. doi: 10.1371/journal.pone.0039053 (PMC3389004; doi:10.1371/journal.pone.0039053)
Supplement: Table S1 — Candidate genes associated with GI-tract survival of L. plantarum WCFS1. (DOCX) [file pone.0039053.s004.docx]

Supplementary table 1. **Candidate genes associated with GI-tract survival of *L. plantarum* WCFS1.**

| ORF^a^ | name | function | Subcellular localization prediction^b^ | Correlation with high survival^c^ | R^2d^ | Importance^e^ | KO / over^f^ | Strain^g^ |
| --- | --- | --- | --- | --- | --- | --- | --- | --- |
| lp_0148 | *lp_0148* | ABC transporter, permease protein, Cobalt (or cobalamine) | Multi-transmembrane | + | 0.523 | 1.156 | over | pNZ3433^h^ |
| lp_0149 | *lp_0149* | ABC transporter, ATP-binding protein, Cobalt (or cobalamine) | Intracellular | + | 0.634 | 1.977 | over | pNZ3433^h^ |
| lp_0217 | *lp_0217* | ABC transporter, permease protein | Multi-transmembrane | - | 0.626 | 2.268 |  |  |
| lp_0315 | *potD* | spermidine/putrescine ABC transporter, substrate binding protein | N-terminally anchored (No CS) | + | 0.348 | 1.001 |  |  |
| lp_0332 | *lp_0332* | extracellular protein (putative) | N-terminally anchored (No CS) | + | 0.555 | 1.001 |  |  |
| lp_0404 | *plnL* | immunity protein PlnL | Multi-transmembrane | + | 0.415 | 1.001 |  |  |
| lp_0415 | *plnA* | plantaricin A precursor peptide, induction factor | Secreted via minor pathways (no CS) | + | 0.147 | 1.001 |  |  |
| lp_0490 | *lp_0490* | unknown | Intracellular | + | 0.155 | 1.001 |  |  |
| lp_0625 | *lp_0625* | prophage P1 protein 2, mitogenic factor, cell surface lipoprotein | Lipid anchored | + | 0.499 | 1.443 |  |  |
| lp_0630 | *lp_0630* | prophage P1 protein 7 | Intracellular | + | 0.498 | 1.001 |  |  |
| lp_0820 | *glmM* | phosphoglucosamine mutase | Intracellular | + | 0.345 | 1.001 |  |  |
| lp_0869 | *lp_0869* | extracellular protein | Secretory (released) (with CS) | - | 0.257 | 1.001 |  |  |
| lp_1188 | *rfbC* | dTDP-4-dehydrorhamnose 3,5-epimerase | Intracellular | + | 0.290 | 1.001 |  |  |
| lp_1189 | *rfbB* | dTDP-glucose 4,6-dehydratase | Intracellular | + | 0.302 | 1.001 |  |  |
| lp_1357 | *lp_1357* | extracellular protein, membrane-anchored (putative) | N-terminally anchored (No CS) | + | 0.233 | 1.001 | over | pNZ3430 |
| lp_1413 | *pbp2A* | transpeptidase-transglycosylase (penicillin binding protein 2A) | N-terminally anchored (No CS) | - | 0.702 | 1.832 | KO | NZ3412^CM^ |
| lp_1515 | *infC* | translation initiation factor IF-3 | Intracellular | - | 0.043 | 1.119 |  |  |
| lp_1562 | *udk* | uridine kinase | Intracellular | + | 0.346 | 1.156 |  |  |
| lp_1669 | *lp_1669* | transcription regulator, AraC family | Intracellular | - | 0.601 | 1.156 | KO | NZ3417^CM^ |
| lp_1817 | *lp_1817* | ribitol-5-phosphate 2-dehydrogenase (putative) | Intracellular | - | 0.378 | 1.156 | KO | NZ3414^CM^ |
| lp_1838 | *lp_1838* | transcription regulator, LysR family | Intracellular | - | 0.009 | 1.092 |  |  |
| lp_1958 | *lp_1958* | acetoin ABC transporter, ATP-binding protein | Intracellular | - | 0.550 | 1.092 |  |  |
| lp_2349 | *hicD3* | L-2-hydroxyisocaproate dehydrogenase | Intracellular | + | 0.441 | 1.001 | over | pNZ3431 |
| lp_2451 | *lp_2451* | prophage P2a protein 6; endonuclease | Intracellular | + | 0.727 | 1.156 |  |  |
| lp_2643 | *lplA1* | lipoate-protein ligase | Intracellular | + | 0.411 | 1.001 |  |  |
| lp_2651 | *lp_2651* | transcription regulator, GntR family | Intracellular | - | 0.334 | 1.001 |  |  |
| lp_2758 | *thrC* | threonine synthase | Intracellular | + | 0.714 | 1.227 | over | pNZ3432 ^i^ |
| lp_2761 | *lp_2761* | O-acetyltransferase | Intracellular | + | 0.432 | 1.688 |  |  |
| lp_2827 | *napA3* | Na(+)/H(+) antiporter | Multi-transmembrane | - | 0.686 | 1.503 | KO | NZ3416^CM^ |
| lp_2960 | *lp_2960* | acyltransferase (putative) |  | + | 0.308 | 1.001 |  |  |
| lp_3019 | *lp_3019* | extracellular protein (putative) | N-terminally anchored (No CS) | - | 0.170 | 1.001 |  |  |
| lp_3296 | *folC2* | folylpolyglutamate synthase / dihydrofolate synthase | Intracellular | + | 0.432 | 1.081 | over | pNZ7026^j^ |
| lp_3297 | *folE* | GTP cyclohydrolase I | Intracellular | + | 0.554 | 1.356 | over | pNZ7026^j^ |
| lp_3299 | *folB* | dihydroneopterin aldolase | Intracellular | + | 0.638 | 1.772 | over | pNZ7026^j^ |
| lp_3398 | *pacL3* | cation transporting P-type ATPase | Multi-transmembrane | - | 0.474 | 1.790 | KO | NZ3415^CM^ |
| lp_3493 | *aroC2* | 3-dehydroquinate dehydratase | Intracellular | + | 0.025 | 1.260 |  |  |
| lp_3661 | *rbsR* | transcription regulator, LacI family, ribose | Intracellular | + | 0.197 | 1.417 |  |  |

a ORF, open reading frame.

b Subcellular localization prediction according to LocateP [Zhou M, Boekhorst J, Francke C, Siezen RJ (2008) LocateP: genome-scale subcellular-location predictor for bacterial proteins. BMC Bioinformatics 9: 173.]

c +, positive correlation; -, negative correlation.

d R^2^ based on linear regression of transcript intensity and GI-tract survival of the eight best and eight worst surviving cultures (see fig 2).

e Importance according to random forest [3].

f KO, knock out; over, overexpression.

g *L. plantarum* KO strains with NZ number or *L. plantarum* strains harboring plasmids (pNZ number).

h pNZ3433 contains *lp­_0148*, *lp­_0149*, and *lp­_0150*.

i pNZ3432 contains *thrC* and *lp_2759*.

j pNZ7026 contains *folB*-*folK*-*folE*-*folC2*-*xtp2*-*folP*.
