# Supplementary material for: Modulation of Lactobacillus plantarum Gastrointestinal Robustness by Fermentation Conditions Enables Identification of Bacterial Robustness Markers
Source: PLoS One. 2012 Jul 3;7(7):e39053. doi: 10.1371/journal.pone.0039053 (PMC3389004; doi:10.1371/journal.pone.0039053)
Supplement: Table S2 — Differentially regulated genes in NZ3417CM (Δ lp_1669::cat ) grown in 2× CDM. (DOCX) [file pone.0039053.s005.docx]

Supplementary table 2. **Differentially regulated genes in NZ3417^CM^ (Δ*lp_1669::cat*) grown in 2**× **CDM.**

| ID | name | function | main class | WCFS1 over Δlp_1669 | fdr WCFS1 over Δlp_1669 |
| --- | --- | --- | --- | --- | --- |
| lp_3688a | *rpmH* | ribosomal protein L34 | Protein synthesis | 158,11 | 0,000 |
| lp_1669 | *lp_1669* | transcription regulator, AraC family | Regulatory functions | 96,30 | 0,002 |
| lp_2901 | *lp_2901* | ABC transporter (putative) | Transport and binding proteins | 15,67 | 0,000 |
| lp_2902 | *lp_2902* | transcription regulator, TetR family | Regulatory functions | 10,36 | 0,000 |
| lp_2903 | *lp_2903* | transcription regulator, ArsR family | Regulatory functions | 7,36 | 0,000 |
| lp_2759 | *lp_2759* | hydrolase, HAD superfamily, Cof family | Hypothetical proteins | 3,71 | 0,004 |
| lp_2760 | *lp_2760* | unknown | Hypothetical proteins | 3,54 | 0,022 |
| lp_2703 | *pyrB* | aspartate carbamoyltransferase | Purines, pyrimidines, nucleosides and nucleotides | 3,46 | 0,002 |
| lp_2702 | *pyrC* | dihydroorotase | Purines, pyrimidines, nucleosides and nucleotides | 3,17 | 0,004 |
| lp_2758 | *thrC* | threonine synthase | Amino acid biosynthesis | 2,92 | 0,016 |
| lp_2701 | *pyrAA* | carbamoyl-phosphate synthase, pyrimidine-specific, small chain | Purines, pyrimidines, nucleosides and nucleotides | 2,77 | 0,010 |
| lp_0295 | *lp_0295* | transport protein, MMPL family | Transport and binding proteins | 2,72 | 0,042 |
| lp_2697 | *pyrE* | orotate phosphoribosyltransferase | Purines, pyrimidines, nucleosides and nucleotides | 2,66 | 0,047 |
| lp_2371 | *pyrP* | uracil transport protein | Transport and binding proteins | 2,35 | 0,031 |
| lp_2700 | *pyrAB* | carbamoyl-phosphate synthase, pyrimidine-specific, large chain | Purines, pyrimidines, nucleosides and nucleotides | 2,32 | 0,039 |
| lp_0416 | *plnB* | histidine protein kinase PlnB; sensor protein | Regulatory functions | 0,44 | 0,036 |
| lp_0538 | *pth* | peptidyl-tRNA hydrolase | Protein synthesis | 0,41 | 0,040 |
| lp_0539 | *mfd* | transcription-repair coupling factor | Transcription | 0,40 | 0,031 |
| lp_0415 | *plnA* | plantaricin A precursor peptide, induction factor | Cellular processes | 0,39 | 0,036 |
| lp_0233 | *mtlD* | mannitol-1-phosphate 5-dehydrogenase | Energy metabolism | 0,39 | 0,023 |
| lp_1210 | *lp_1210* | unknown | Hypothetical proteins | 0,38 | 0,023 |
| lp_3060 | *lp_3060* | transcription regulator, AraC family | Regulatory functions | 0,37 | 0,032 |
| lp_2515 | *lp_2515* | phosphohydrolase, MutT/nudix family (putative) | Purines, pyrimidines, nucleosides and nucleotides | 0,34 | 0,012 |
| lp_0258 | *lp_0258* | unknown | Hypothetical proteins | 0,33 | 0,018 |
| lp_0285 | *lp_0285* | transcription regulator, RpiR family | Regulatory functions | 0,31 | 0,016 |
| lp_0232 | *pts2A* | mannitol PTS, EIIA | Transport and binding proteins | 0,31 | 0,026 |
| lp_2600 | *tal1* | transaldolase | Energy metabolism | 0,28 | 0,033 |
| lp_0231 | *mtlR* | transcription regulator, mannitol operon | Regulatory functions | 0,27 | 0,020 |
| lp_2551 | *hisC* | histidinol-phosphate aminotransferase | Amino acid biosynthesis | 0,25 | 0,004 |
| lp_2598 | *pflF* | formate C-acetyltransferase (similar to) | Energy metabolism | 0,25 | 0,046 |
| lp_2552 | *hisE* | phosphoribosyl-ATP pyrophosphatase | Amino acid biosynthesis | 0,23 | 0,001 |
| lp_2559 | *hisD* | histidinol dehydrogenase | Amino acid biosynthesis | 0,22 | 0,001 |
| lp_2599 | *lp_2599* | transcription regulator, DeoR family | Regulatory functions | 0,22 | 0,001 |
| lp_3346 | *lp_3346* | unknown | Hypothetical proteins | 0,20 | 0,000 |
| lp_3678 | *lp_3678* | cell surface protein precursor, DUF916 family | Cell envelope | 0,19 | 0,035 |
| lp_2557 | *hisH* | imidazole glycerol phosphate synthase, amidotransferase sununit | Amino acid biosynthesis | 0,18 | 0,002 |
| lp_2554 | *hisF* | imidazoleglycerol phosphate synthase, cyclase subunit | Amino acid biosynthesis | 0,18 | 0,000 |
| lp_2757 | *lp_2757* | maltogenic alpha-amylase | Central intermediary metabolism | 0,17 | 0,008 |
| lp_2553 | *hisI* | phosphoribosyl-AMP cyclohydrolase | Amino acid biosynthesis | 0,17 | 0,000 |
| lp_1652 | *trpE* | anthranilate synthase, component I | Amino acid biosynthesis | 0,16 | 0,022 |
| lp_2556 | *hisA* | phosphoribosylformimino-5-aminoimidazole carboxamideribotide isomerase | Amino acid biosynthesis | 0,15 | 0,002 |
| lp_1207 | *cps2K* | polysaccharide biosynthesis protein (putative) | Cell envelope | 0,15 | 0,000 |
| lp_2560 | *hisG* | ATP phosphoribosyltransferase | Amino acid biosynthesis | 0,14 | 0,002 |
| lp_1206 | *cps2J* | glycosyltransferase | Cell envelope | 0,14 | 0,000 |
| lp_2561 | *hisX* | histidine--tRNA ligase (putative) | Protein synthesis | 0,12 | 0,000 |
| lp_1657 | *trpA* | tryptophan synthase, beta chain | Amino acid biosynthesis | 0,11 | 0,018 |
| lp_3117 | *lp_3117* | cell surface protein (putative) | Cell envelope | 0,11 | 0,047 |
| lp_3074 | *lp_3074* | cell surface protein precursor | Cell envelope | 0,11 | 0,043 |
| lp_3345 | *spx4* | regulatory protein Spx | Regulatory functions | 0,09 | 0,000 |
| lp_2558 | *hisB* | imidazoleglycerol-phosphate dehydratase | Amino acid biosynthesis | 0,08 | 0,001 |
| lp_1205 | *cps2I* | oligosaccharide transporter (flippase) | Cell envelope | 0,08 | 0,000 |
| lp_1204 | *cps2H* | polysaccharide polymerase | Cell envelope | 0,07 | 0,000 |
| lp_1525 | *lp_1525* | integral membrane protein | Hypothetical proteins | 0,07 | 0,000 |
| lp_1203 | *cps2G* | polysaccharide biosynthesis protein | Cell envelope | 0,06 | 0,000 |
| lp_1200 | *cps2D* | UDP N-acetyl glucosamine 4-epimerase, NAD dependent | Purines, pyrimidines, nucleosides and nucleotides | 0,05 | 0,000 |
| lp_1524 | *ica1* | glycosyltransferase | Cell envelope | 0,05 | 0,001 |
| lp_1201 | *cps2E* | priming glycosyltransferase | Cell envelope | 0,05 | 0,000 |
| lp_1202 | *cps2F* | glycosyltransferase | Cell envelope | 0,04 | 0,000 |
| lp_1198 | *cps2B* | polysaccharide biosynthesis protein; regulator | Cell envelope | 0,03 | 0,000 |
| lp_1199 | *cps2C* | polysaccharide biosynthesis protein; phosphatase (putative) | Cell envelope | 0,03 | 0,000 |
| lp_1197 | *cps2A* | polysaccharide biosynthesis protein, chain length regulator (putative) | Cell envelope | 0,02 | 0,000 |
| lp_0498 | *deoP* | deoxyribose transporter | Transport and binding proteins | 0,00 | 0,000 |
