# Supplementary material for: Modulation of Lactobacillus plantarum Gastrointestinal Robustness by Fermentation Conditions Enables Identification of Bacterial Robustness Markers
Source: PLoS One. 2012 Jul 3;7(7):e39053. doi: 10.1371/journal.pone.0039053 (PMC3389004; doi:10.1371/journal.pone.0039053)
Supplement: Table S3 — Differentially regulated genes in NZ3417CM (Δ lp_1669::cat ) grown in MRS. (DOCX) [file pone.0039053.s006.docx]

Supplementary table 3. **Differentially regulated genes in NZ3417^CM^ (Δ*lp_1669::cat*) grown in** **MRS.**

| ID | name | function | main class | WCFS1 over Δlp_1669 | fdr WCFS1 over Δlp_1669 |
| --- | --- | --- | --- | --- | --- |
| lp_1669 | *lp_1669* | transcription regulator, AraC family | Regulatory functions | 990,99 | 0,000 |
| lp_2759 | *lp_2759* | hydrolase, HAD superfamily, Cof family | Hypothetical proteins | 3,20 | 0,006 |
| lp_2760 | *lp_2760* | unknown | Hypothetical proteins | 2,93 | 0,004 |
| lp_2758 | *thrC* | threonine synthase | Amino acid biosynthesis | 2,79 | 0,000 |
| lp_1219 | *glf2* | UDP-galactopyranose mutase | Purines, pyrimidines, nucleosides and nucleotides | 0,50 | 0,001 |
| lp_3678 | *lp_3678* | cell surface protein precursor, DUF916 family | Cell envelope | 0,49 | 0,014 |
| lp_3060 | *lp_3060* | transcription regulator, AraC family | Regulatory functions | 0,49 | 0,005 |
| lp_3520 | *pts32A* | glucose PTS, EIIA | Transport and binding proteins | 0,49 | 0,025 |
| lp_0579 | *panD* | aspartate 1-decarboxylase | Energy metabolism | 0,48 | 0,034 |
| lp_1221 | *cps3E* | polysaccharide biosynthesis protein (putative) | Cell envelope | 0,47 | 0,024 |
| lp_2341 | *bla2* | beta-lactamase (putative) | Cell envelope | 0,47 | 0,005 |
| lp_1227 | *cps3J* | glycosyltransferase | Cell envelope | 0,46 | 0,025 |
| lp_p3_15 |  |  |  | 0,46 | 0,017 |
| lp_2226 | *lp_2226* | competence protein/transcription factor, CoiA-like family | Cellular processes | 0,45 | 0,027 |
| lp_3521 | *lp_3521* | transcription regulator, RpiR family | Regulatory functions | 0,45 | 0,005 |
| lp_0538 | *pth* | peptidyl-tRNA hydrolase | Protein synthesis | 0,44 | 0,004 |
| lp_0286 | *pts6C* | cellobiose PTS, EIIC | Transport and binding proteins | 0,43 | 0,002 |
| lp_3570 | *lp_3570* | transposase (putative) | Other categories | 0,43 | 0,048 |
| lp_2108 | *cps4A* | polysaccharide biosynthesis protein, chain length regulator (putative) | Cell envelope | 0,42 | 0,012 |
| lp_0664 | *lp_0664* | prophage P1 protein 41, scaffold protein | Other categories | 0,42 | 0,017 |
| lp_3348 | *lp_3348* | unknown | Hypothetical proteins | 0,41 | 0,002 |
| lp_0231 | *mtlR* | transcription regulator, mannitol operon | Regulatory functions | 0,40 | 0,012 |
| lp_1220 | *cps3D* | polysaccharide biosynthesis protein (putative) | Cell envelope | 0,40 | 0,001 |
| lp_0285 | *lp_0285* | transcription regulator, RpiR family | Regulatory functions | 0,40 | 0,017 |
| lp_0472 | *lp_0472* | integral membrane protein | Hypothetical proteins | 0,40 | 0,001 |
| lp_0297 | *lp_0297* | extracellular protein | Cell envelope | 0,40 | 0,013 |
| lp_1207 | *cps2K* | polysaccharide biosynthesis protein (putative) | Cell envelope | 0,38 | 0,001 |
| lp_p3_17 |  |  |  | 0,37 | 0,009 |
| lp_3135 | *tkt3* | transketolase | Energy metabolism | 0,37 | 0,010 |
| lp_2600 | *tal1* | transaldolase | Energy metabolism | 0,36 | 0,033 |
| lp_1908 | *lp_1908* | integral membrane protein | Hypothetical proteins | 0,36 | 0,000 |
| lp_1222 | *cps3F* | polysaccharide polymerase | Cell envelope | 0,36 | 0,003 |
| lp_2635 | *lp_2635* | integral membrane protein | Hypothetical proteins | 0,36 | 0,017 |
| lp_1215 | *cps3A* | glycosyltransferase | Cell envelope | 0,35 | 0,018 |
| lp_2230 | *lp_2230* | unknown | Hypothetical proteins | 0,35 | 0,012 |
| lp_0139 | *lai* | linoleic acid isomerase | Cell envelope | 0,35 | 0,001 |
| lp_0233 | *mtlD* | mannitol-1-phosphate 5-dehydrogenase | Energy metabolism | 0,34 | 0,001 |
| lp_2812 | *lp_2812* | extracellular protein, membrane-anchored (putative) | Cell envelope | 0,32 | 0,002 |
| lp_2515 | *lp_2515* | phosphohydrolase, MutT/nudix family (putative) | Purines, pyrimidines, nucleosides and nucleotides | 0,31 | 0,001 |
| lp_2106 | *cps4C* | polysaccharide biosynthesis protein; phosphatase (putative) | Cell envelope | 0,30 | 0,001 |
| lp_0232 | *pts2A* | mannitol PTS, EIIA | Transport and binding proteins | 0,30 | 0,001 |
| lp_0230 | *pts2CB* | mannitol PTS, EIICB | Transport and binding proteins | 0,29 | 0,040 |
| lp_0500 | *deoK* | deoxyribokinase | Energy metabolism | 0,29 | 0,042 |
| lp_2598 | *pflF* | formate C-acetyltransferase (similar to) | Energy metabolism | 0,29 | 0,012 |
| lp_1206 | *cps2J* | glycosyltransferase | Cell envelope | 0,29 | 0,000 |
| lp_0365 | *lp_0365* | integral membrane protein | Hypothetical proteins | 0,29 | 0,012 |
| lp_2103 | *cps4F* | glycosyltransferase | Cell envelope | 0,28 | 0,004 |
| lp_1225 | *cps3H* | polysaccharide biosynthesis protein (putative) | Cell envelope | 0,27 | 0,001 |
| lp_2107 | *cps4B* | exopolysaccharide biosynthesis protein | Cell envelope | 0,27 | 0,000 |
| lp_1205 | *cps2I* | oligosaccharide transporter (flippase) | Cell envelope | 0,25 | 0,001 |
| lp_2105 | *cps4D* | UDP N-acetyl glucosamine 4-epimerase, NAD dependent | Purines, pyrimidines, nucleosides and nucleotides | 0,25 | 0,000 |
| lp_1235 | *lp_1235* | unknown | Hypothetical proteins | 0,24 | 0,029 |
| lp_2100 | *cps4I* | glycosyltransferase | Cell envelope | 0,24 | 0,000 |
| lp_2104 | *cps4E* | priming glycosyltransferase | Cell envelope | 0,23 | 0,001 |
| lp_2101 | *cps4H* | polysaccharide polymerase | Cell envelope | 0,22 | 0,000 |
| lp_3346 | *lp_3346* | unknown | Hypothetical proteins | 0,22 | 0,000 |
| lp_2102 | *cps4G* | glycosyltransferase | Cell envelope | 0,20 | 0,002 |
| lp_3136 | *lp_3136* | sugar-specific permease | Transport and binding proteins | 0,19 | 0,001 |
| lp_3127 | *lp_3127* | mucus-binding protein (putative) | Cell envelope | 0,19 | 0,048 |
| lp_3074 | *lp_3074* | cell surface protein precursor | Cell envelope | 0,19 | 0,000 |
| lp_3657 | *srlD2* | sorbitol-6-phosphate 2-dehydrogenase | Energy metabolism | 0,17 | 0,019 |
| lp_3138 | *lp_3138* | bifunctional protein: transcriptional antiterminator, BglG family; PTS, EIIA | Transport and binding proteins | 0,17 | 0,033 |
| lp_3115 | *lp_3115* | cell surface protein precursor, DUF916 family | Cell envelope | 0,17 | 0,033 |
| lp_3116 | *lp_3116* | extracellular protein | Cell envelope | 0,16 | 0,006 |
| lp_0298 | *lp_0298* | ABC transporter, permease protein | Transport and binding proteins | 0,15 | 0,031 |
| lp_2757 | *lp_2757* | maltogenic alpha-amylase | Central intermediary metabolism | 0,14 | 0,000 |
| lp_2796 | *lp_2796* | cell surface protein precursor | Cell envelope | 0,14 | 0,010 |
| lp_1204 | *cps2H* | polysaccharide polymerase | Cell envelope | 0,14 | 0,000 |
| lp_1202 | *cps2F* | glycosyltransferase | Cell envelope | 0,13 | 0,000 |
| lp_2099 | *cps4J* | repeat unit transporter (flippase) | Cell envelope | 0,11 | 0,000 |
| lp_3073 | *lp_3073* | extracellular protein | Cell envelope | 0,11 | 0,011 |
| lp_1200 | *cps2D* | UDP N-acetyl glucosamine 4-epimerase, NAD dependent | Purines, pyrimidines, nucleosides and nucleotides | 0,11 | 0,000 |
| lp_2599 | *lp_2599* | transcription regulator, DeoR family | Regulatory functions | 0,11 | 0,000 |
| lp_1201 | *cps2E* | priming glycosyltransferase | Cell envelope | 0,11 | 0,000 |
| lp_1525 | *lp_1525* | integral membrane protein | Hypothetical proteins | 0,09 | 0,000 |
| lp_1203 | *cps2G* | polysaccharide biosynthesis protein | Cell envelope | 0,09 | 0,000 |
| lp_3117 | *lp_3117* | cell surface protein (putative) | Cell envelope | 0,09 | 0,032 |
| lp_3476 | *ramR* | transcription regulator, AraC family, GlcNAc-like induced | Regulatory functions | 0,09 | 0,049 |
| lp_3345 | *spx4* | regulatory protein Spx | Regulatory functions | 0,09 | 0,000 |
| lp_3278 | *lp_3278* | amino acid transport protein | Transport and binding proteins | 0,06 | 0,033 |
| lp_1198 | *cps2B* | polysaccharide biosynthesis protein; regulator | Cell envelope | 0,06 | 0,000 |
| lp_3646 | *lp_3646* | transcription regulator, AraC family | Regulatory functions | 0,06 | 0,008 |
| lp_1396 | *lp_1396* | transcription regulator, LuxR family | Regulatory functions | 0,06 | 0,012 |
| lp_0250 | *lp_0250* | unknown | Hypothetical proteins | 0,05 | 0,000 |
| lp_1524 | *ica1* | glycosyltransferase | Cell envelope | 0,05 | 0,001 |
| lp_1199 | *cps2C* | polysaccharide biosynthesis protein; phosphatase (putative) | Cell envelope | 0,04 | 0,000 |
| lp_1197 | *cps2A* | polysaccharide biosynthesis protein, chain length regulator (putative) | Cell envelope | 0,04 | 0,000 |
