# Supplementary material for: Modulation of Lactobacillus plantarum Gastrointestinal Robustness by Fermentation Conditions Enables Identification of Bacterial Robustness Markers
Source: PLoS One. 2012 Jul 3;7(7):e39053. doi: 10.1371/journal.pone.0039053 (PMC3389004; doi:10.1371/journal.pone.0039053)
Supplement: Table S4 — Primers used in this study. (DOCX) [file pone.0039053.s007.docx]

Supplementary table 4. **Primers used in this study.**

| **ID** | **Name** | **Sequence (5’ to 3’)^a,b^** | **Reference** |
| --- | --- | --- | --- |
| A1 | pbp2A-outI | AGTTCTGTGCGTAGTTTGCC | This work |
| A2 | pbp2A-1412F | TTTGCTATAATGTATTCATTAC | This work |
| A3 | ppbp2A-1412R | gcatacattatacgaacggtagatttTTTTTGCATAATCTTCCCCTTGTTCAGC | This work |
| A4 | pbp2A-1414F | cggttacagcccgggcatgagTAGTAAAGCTAGCTTCTGAACG | This work |
| A5 | pbp2A-1414R | GACCGTGCAAGGTACCAATC | This work |
| A6 | pbp2A-outII | TAGTGGTCACCCGCCACACC | This work |
| B1 | lp-1669-outI | ATCATGGCTTAATCAACAGCG | This work |
| B2 | lp-1669-1668F | CGCCAGGCGTAATGAGTGTG | This work |
| B3 | lp-1669-1668R-inverted cat | gcatacattatacgaacggtagatttAATCTTCACACTAATCACTCCTAC | This work |
| B4 | lp-1669-1670F-inverted cat | cggttacagcccgggcatgagTAACAAGCGTTGCCGTTTAGG | This work |
| B5 | lp-1669-1670R | CGAAAAATTAGTTGTCATGG | This work |
| B6 | lp-1669-outII | AAATTAGTTGTCATGGTTGG | This work |
| C1 | lp-1817-outI | CGCGACAGAGAAGTCCAACC | This work |
| C2 | lp-1817-1816F | TTTCGTAGACGAGTCAAAG | This work |
| C3 | lp-1817-1816R | gcatacattatacgaacggtagatttATTTAACATCTTATGACCTCTTTTTC | This work |
| C4 | lp-1817-1818F | cggttacagcccgggcatgagTAAAGACGGTAAAGCTCGTGTTAC | This work |
| C5 | lp-1817-1818R | ATATGATCAACTTCCTGATT | This work |
| C6 | lp-1817-outII | CATGTACATAAGATAGATCC | This work |
| D1 | pacL3-outI | GGTAATCATAGCAACATTAG | This work |
| D2 | pacL3-3397F | CATACCAGGTTGTGTCACGG | This work |
| D3 | pacL3-3397R | gcatacattatacgaacggtagatttATTCTGCATCGTTTATTCCGTAATTCG | This work |
| D4 | pacL3-3399F | cggttacagcccgggcatgagTAAGGATGATCAATTCAAGTTAGTTAAAATG | This work |
| D5 | pacL3-3399R | GTTGATTAACAAAATTACTG | This work |
| D6 | pacL3-outII | TCAATATCATTTTCAGTTTG | This work |
| E1 | napA3-outI | AGTCTGGGCATGCATGAAGC | This work |
| E2 | napA3-2826F | AACGAGCAGGCCGACGAGC | This work |
| E3 | napA3-2826R | gcatacattatacgaacggtagatttGTAATCCATTAAAAACCTCCTAAAAAAGG | This work |
| E4 | napA3-2828F | cggttacagcccgggcatgagTAAAGCAATTGAAAATCCCAACTTG | This work |
| E5 | napA3-2828R | TCCTGGGAAGTTTACGAACC | This work |
| E6 | napA3-outII | CCGATAACTGAAGTTCTTGG | This work |
| F1 | lp-1357-overexpression F | CCCCCTCATGAAGCAGTTCTGGTCACTAATC | This work |
| F2 | lp-1357-overexpression R | CTAACTCTTTGTCCCGGTTGG | This work |
| G1 | hicD3-overexpression F | CCCCCCCATGGCTCGTAAATATGGTGTGATCGGG | This work |
| G2 | hicD3-overexpression R | TTATGCTTGCGGTAAAACGTCC | This work |
| H1 | thrC+lp-2759 overexpression F | CCCCCTCATGAAAACACTTTATCGCAGTACC | This work |
| H2 | thrC+lp-2759 overexpression R | TCAGTTGAAGTAATTTTCTAGGAAAA | This work |
| I1 | lp-0148~0150 overexpression F | CCCCCACATGTCTCAAAACAAGCAATCCAATTCAATTCG | This work |
| I2 | lp-0148~0150 overexpression R | TTATGCCTTAAACGGATTCCAG | This work |
| I | TAG-lox66-F2 | CGGGAGCAGAATGTCCGAGACTAATG | [1]^c^ |
| J | TAG-lox71-catR2 | TAGTGCGTCTTCTCGTAGCGATCGG | [1] |
| R87 | 87 | GCCGACTGTACTTTCGGATCC | [2] |
| Is169 | 169 | TTATCATATCCCGAGGACCG | This work |
| S1-2 | Sequencing primer R of pSIP411 | GTAATTGCTTTATCAACTGCTGC | This work |
| S2-3 | Sequencing primer 3 of thrC+lp-2759 | ACCATACTTACAACAACTTGAACTCAACC | This work |
| S3-4 | Sequencing primer 4 of lp-0148~0150 | GATCTCTACAACGATGATTTTTGATGAAG | This work |

^a^ The lower-case letters indicates the overhang sequences that homolgous to the ultimate regions of the *cat* (chloramphenicol acetyltransferase) amplicon.

^b^ Underlined are the restriction sites.

^c^ References:

1. van Bokhorst-van de Veen H, Abee T, Tempelaars M, Bron PA, Kleerebezem M, et al. (2011) Short- and long-term adaptation to ethanol stress and its cross-protective consequences in *Lactobacillus plantarum*. Appl Environ Microbiol 77: 5247-5256.

2. Lambert JM, Bongers RS, Kleerebezem M (2007) Cre-*lox*-based system for multiple gene deletions and selectable-marker removal in *Lactobacillus plantarum*. Appl Environ Microbiol 73: 1126-1135.
