# Supplementary material for: Modulation of Lactobacillus plantarum Gastrointestinal Robustness by Fermentation Conditions Enables Identification of Bacterial Robustness Markers
Source: PLoS One. 2012 Jul 3;7(7):e39053. doi: 10.1371/journal.pone.0039053 (PMC3389004; doi:10.1371/journal.pone.0039053)
Supplement: Table S5 — Primer pair combinations used for LF and RF amplification and for the SOE step of the deletion mutants. (DOCX) [file pone.0039053.s008.docx]

Supplementary table 5. **Primer pair combinations used for LF and RF amplification and for the SOE step of the deletion mutants.**

| Label | Target gene | Left flank primer pair | Right flank primer pair | SOE primer pair |
| --- | --- | --- | --- | --- |
| A | *pbp2A* | A2 / A3 | A4 / A5 | A2 / A5 |
| B | *lp-1669* | B2 / B3 | B4 / B5 | B2 / B5 |
| C | *lp-1817* | C2 / C3 | C4 / C5 | C2 / C5 |
| D | *pacL3* | D2 / D3 | D4 / D5 | D2 / D5 |
| E | *napA3* | E2 / E3 | E4 / E5 | E2 / E5 |
