# Supplementary material for: Modulation of Lactobacillus plantarum Gastrointestinal Robustness by Fermentation Conditions Enables Identification of Bacterial Robustness Markers
Source: PLoS One. 2012 Jul 3;7(7):e39053. doi: 10.1371/journal.pone.0039053 (PMC3389004; doi:10.1371/journal.pone.0039053)
Supplement: Table S6 — Primer pair combinations used for each deletion mutant to confirm the correct integration in the genome. (DOCX) [file pone.0039053.s009.docx]

Supplementary table 6. **Primer pair combinations used for each deletion mutant to confirm the correct integration in the genome.**

| Label | Target gene | Left side | Right side |
| --- | --- | --- | --- |
| A | *pbp2A* | A1 / Is169 | R87 / A6 |
| B | *lp-1669* | B1 / R87 | Is169 / B6 |
| C | *lp-1817* | C1 / Is169 | R87 / C6 |
| D | *pacL3* | D1 / Is169 | R87 / D6 |
| E | *napA3* | E1 / Is169 | R87 / E6 |
